# New CXCR1/CXCR2 inhibitors represent an effective treatment for kidney or head and neck cancers sensitive or refractory to reference treatments

**DOI:** 10.7150/thno.34681

**Published:** 2019-07-09

**Authors:** Maeva Dufies, Oleksandr Grytsai, Cyril Ronco, Oumar Camara, Damien Ambrosetti, Anaïs Hagege, Julien Parola, Lou Mateo, Marion Ayrault, Sandy Giuliano, Renaud Grépin, Nathalie Lagarde, Matthieu Montes, Patrick Auberger, Luc Demange, Rachid Benhida, Gilles Pagès

**Affiliations:** 1 Centre Scientifique de Monaco, Biomedical Department, Principality of Monaco.; 2 Université Côte d'Azur, CNRS, Institut de Chimie de Nice UMR 7272, 06108, Nice, France.; 3 Université Côte d'Azur, Centre Hospitalier Universitaire (CHU) de Nice, Hôpital Pasteur, Department of Pathology, Nice, France.; 4 Université Côte d'Azur, CNRS UMR 7284 and INSERM U 1081, Institute for Research on Cancer and Aging (IRCAN), 28 Avenue de Valombrose, 06107 Nice, France.; 5 Centre Antoine Lacassagne, Nice, France.; 6 Laboratoire GBCM EA7528, Conservatoire National des Arts et Métiers, 2 Rue Conté, 75003 Paris, France.; 7 Université Côte d'Azur, INSERM U1065, Centre Méditerranéen de Médecine Moléculaire (C3M), Bâtiment ARCHIMED, 151 Route de Saint-Antoine de Ginestière, BP 2 3194, 06204 Nice Cedex 3, France.; 8 Université de Paris, CiTCoM, UMR 8038 CNRS, F-75006 Paris, France.; 9 Mohamed VI Polytechnic University, UM6P, 43150 BenGuerir, Morocco.

**Keywords:** ELR^+^CXCL cytokines, Clear cell Renal Cell Carcinoma, Head and Neck Squamous Cell Carcinoma, CXCR1/2 inhibitor, angiogenesis.

## Abstract

Clear cell Renal Cell (RCC) and Head and Neck Squamous Cell Carcinomas (HNSCC) are characterized by a pro-angiogenic/pro-inflammatory context. Despite conventional or targeted therapies, metastatic RCC and HNSCC remain incurable. Alternative treatments to reference therapies (sunitinib, a multi tyrosine kinase inhibitor for RCC or cisplatin for HNSCC) are urgently needed on relapse. Here, we described the relevance of targeting the ELR+CXCL cytokines receptors, CXCR1/2, for the treatment of these two cancer types.

**Methods**: The relevance to patient treatment was evaluated by correlating the ELR^+^CXCL/CXCR1/2 levels to survival using online available data. We report herein the synthesis of new pharmacological inhibitors of CXCR1/2 with anti-proliferation/survival activity. The latter was evaluated with the XTT assay with leukemic, breast, RCC and HNSCC cell lines. Their relevance as an alternative treatment was tested on sunitinib- and cisplatin- resistant cells. The most efficient compound was then tested in a mouse model of RCC and HNSCC.

**Results**: RCC and HNSCC expressed the highest amounts of CXCR1/2 of all cancers. High levels of ELR^+^CXCL cytokines (CXCL1, 2, 3, 5, 6, 7, 8) correlated to shorter survival. Among the 33 synthesized and tested molecules, compound C29 reduced ELR^+^CXCL/CXCR1/2-dependent proliferation and migration of endothelial cells. C29 exerted an anti-proliferation/survival activity on a panel of cancer cells including naive and resistant RCC and HNSCC cells. C29 reduced the growth of experimental RCC and HNSCC tumors by decreasing tumor cell proliferation, angiogenesis and ELR^+^/CXCL-mediated inflammation.

**Conclusion**: Our study highlights the relevance of new CXCR1/2 inhibitors for the treatment of RCC or HNSCC as first-line treatment or at relapse on reference therapies.

## Introduction

Angiogenesis and inflammation are two closely interconnected hallmarks of cancer 1. Inflammatory conditions induce the production of vascular endothelial growth factor (VEGF) by endothelial cells, tumor associated fibroblasts and tumor cells, resulting in angiogenesis. Hyper vascularization favors the transport of inflammatory cells and acute permeability of the neo-formed vessels facilitates the development of edema. Overall, inflammation induces angiogenesis, and angiogenesis enhances inflammation 2, 3.

Therefore, we aimed at developing new molecules to tackle concomitantly these two phenomena. In this study, we focused our attention on the pro-angiogenic and pro-inflammatory ELR^+^CXCL cytokines that include CXCL1-3, 5-8. These cytokines bind to two seven-transmembrane heptahelical G protein-coupled receptors, namely CXCR1 and CXCR2. Receptor stimulation activates several signaling pathways including the protein kinase C, phospholipase C, PI3K/AKT/mTOR, RAS/RAF/MEK/ERK and NFĸB pathways, leading to tumor cell survival, proliferation, and dissemination 4.

The leading member of the ELR^+^CXCL cytokines is CXCL8 (also called interleukin 8 or IL-8), which promotes angiogenesis, tumorigenesis, and metastasis 5. The levels also correlated with tumor burden in several cancer types including prostate, ovarian, brain, and skin cancers 6. CXCL1 is involved in esophageal, gastric, colorectal and skin cancer cell proliferation 7-10. CXCL7 is a key player in the development of renal cell carcinoma (RCC), and in its response to sunitinib 11, 12. Lastly, CXCL5 and CXCL8 correlated to head and neck squamous cell carcinoma (HNSCC) aggressiveness 13, 14. CXCR1/2 are expressed by endothelial and inflammatory cells and are key players in angiogenesis and inflammation, especially in a hypoxic environment 15. Moreover, the intra-tumor expression of CXCR2 correlated with relapse and poor prognosis of RCC patients with non-metastatic disease (M0) 16.

Thus, we hypothesized that small-sized organic antagonists of the ELR^+^CXCL that bind to CXCR1/2 might exert a dual activity on both angiogenesis and inflammation. CXCR1/2 competitive and non-competitive inhibitors have been developed mainly for the treatment of pulmonary inflammatory disorders, and recently for advanced metastatic breast cancers 17.

We previously reported that the competitive CXCR1/2 inhibitor, SB225002, delays *in vivo* tumor growth and inflammation by antagonizing the signaling pathways induced by CXCL7. However, the anti-proliferative effect of SB225002 remains modest *in vitro* 12. Therefore, the investigation of more potent analogues is of utmost interest. In line with these observations, two series of new and original *N*, *N*'-diaryl ureas and thioureas, featuring a nitro-benzothiazole moiety, were synthesized in an efficient way and evaluated as potential anti-cancer agents in two deadly cancers RCC and HNSCC. Among the 33 synthesized molecules, compound C29 emerged as a promising lead, with interesting dual anti-angiogenic and anti-proliferative activities. Moreover, C29 reduced significantly the growth of experimental tumors in mice. Therefore, this study paves the way for future clinical trials targeting a pathway leading to three major hallmarks of cancers, *i.e.,* tumor cell proliferation, angiogenesis and inflammation.

## Methods

### Chemistry

**Figure 1:**
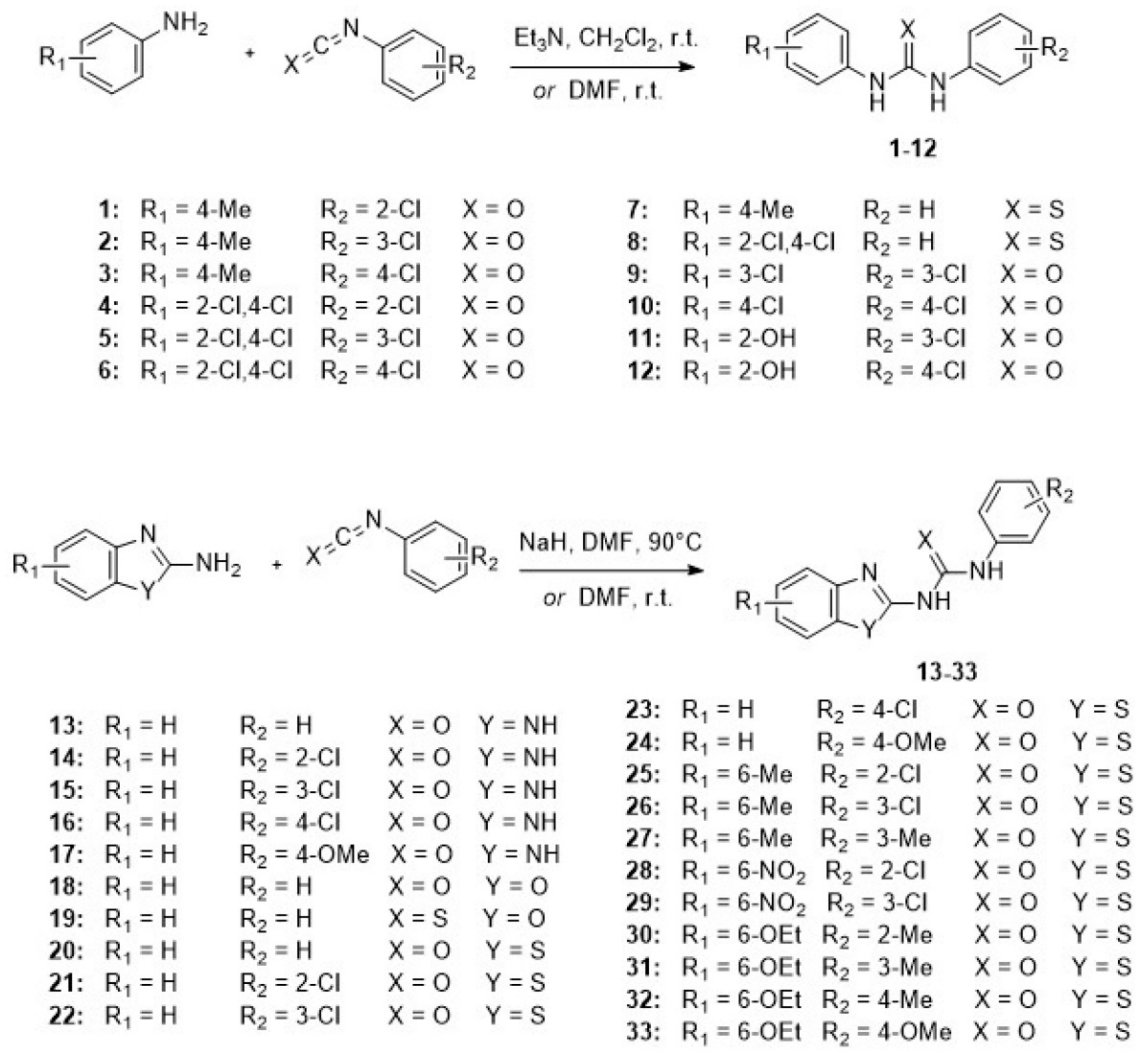
**CXCR1/CXCR2 inhibitors**. Chemical structures of the synthesized *N*, *N*'-diarylureas and -thioureas, that have been evaluated as potential CXCR1 antagonists.

The *N*, *N*'-diarylureas and thioureas (C1-C12) were conveniently synthesized according to a one-step procedure (**Figure 1 and Figure S1**), that should be used on a large scale for *in vivo* tests and pilot extrapolation. Briefly, the reaction consists in the nucleophilic attack of substituted anilines on mono- or di-substituted isocyanates and isothiocyanates, followed by spontaneous tautomerization. The expected *N*,*N*'-disubstituted ureas and thioureas have been obtained in good yields within a short reaction time by simple mixing of reactants at room temperature in the presence of an organic base (**Figure 1, s**ee also Supplementary Methods for details).

### Molecular simulation studies

The RMN structure of CXCR1 (PDB ID : 2LNL) was retrieved from the Protein Data Bank (PDB) 18. The RMN structure includes 10 conformations for CXCR1. Each conformation was considered separately, and the 10 resulting CXCR1 structures were prepared using MGL tools 19. Three-dimensional structures of compound C29 were generated using iCon, the LigandScout v.4.3 conformer generator 20 (defaults settings of the BEST option were used, except for the maximum number of conformations generated that was set to 50 instead of 25). A total of 22 conformers of compound C29 was obtained. Protein - ligand docking of compound C29 into the CXCR1 structure was performed using AutoDock Vina v.1.1.2 21. As no information about the CXCR1 binding site of compound C29 was available, a blind docking approach was selected by docking compound C29 to the whole surface of CXCR1, without restriction of the search space. Each of the 22 conformers of compound C29 were docked into each of the 10 CXCR1 structures, resulting in 220 docking runs. For each run, 9 poses of compound C29 bound to CXCR1 were generated. The pose associated with the best score was considered for each run.

### Reagents and antibodies

Sunitinib, SB225002 and danirixin were purchased from Selleckchem. Anti-HSP60 antibodies were purchased from Santa Cruz Biotechnology. Anti-AKT, anti-phospho-AKT, anti-ERK, anti-phospho-ERK antibodies were from Cell Signaling Technology. Methanol, ethyl acetate, diethyl ether and dichloromethane were purchased from Carlo Erba. Anhydrous DMF was purchased from Sigma Aldrich. All chemicals were purchased from Aldrich, Fisher or Alfa Aesar and used without further purification.

### Cell culture

RCC4, 786-0 (786) and A498 (498) RCC cell lines, the MDA-MD-231 breast cancer cell line were purchased from the American Tissue Culture Collection. OCI-AML2, OCI-AML3, Molm13 and Molm14 acute myeloid cell lines (AML), and K562 chronic myeloid cell line (CML), SKM1 myelodysplastic cell line (MDS) were a kind gift from Dr. P. Auberger. Two human HNSCC cell lines, CAL33 and CAL27, were provided through a Material Transfer Agreement with the Oncopharmacology Laboratory, Centre Antoine Lacassagne (CAL) where they were initially isolated 22. Primary RCC cells (CC, TF and 15S) have already been described and cultured in a medium specific for renal cells (PromoCell) 23.

### Immunoblotting

Cells were lysed in buffer containing 3% SDS, 10% glycerol and 0.825 mM Na_2_HPO_4_. 30 to 50 μg of proteins separated on 10% SDS-PAGE, transferred onto a PVDF membrane and then exposed to the appropriate antibodies. Proteins were visualized with the ECL system using horseradish peroxidase-conjugated anti-rabbit or anti-mouse antibodies.

### Migration assay

CXCL7 or VEGFA-stimulated chemotaxis assays were monitored using modified Boyden chambers containing polycarbonate membranes (8-μm pores, Transwell; Corning, Sigma). Cells were seeded onto the upper side of the filters and chambers were placed on 24-well plates containing CXCL7 (50ng/ml) or VEGFA (50ng/ml). Cell migration was followed for 24 h at 37°C in 5% CO_2_. Migratory cells on the lower membrane surface were fixed in 3% paraformaldehyde, stained with 0.1% crystal violet.

### Colony formation assay

Cells (5000 cells per condition) were treated or not with C29, sunitinib. Colonies were detected after 10 days of culture. Cells were then washed, fixed at room temperature for 20 min with 3% paraformaldehyde (PFA; Electron Microscopy Sciences) and colored with GIEMSA (Sigma).

### Caspase assays

Caspase assays have already been described 24. Briefly, the caspase 3 activity was assessed in quadruplicate using z-DEVD-AMC as substrate and the fluorescence were assessed.

### Flow cytometry

*CXCR2 measurement*: After stimulation, cells were washed with PBS and stained with the CXCR2-PE antibody (Miltenyi) for 30 min at room temperature. Fluorescence was measured using the FL2 (PE) channels of a fluorescence-activated cell sorter apparatus (Calibur cytometer).

*Apoptosis analysis*: After stimulation, cells were washed with ice-cold PBS and were stained with the annexin-V-fluo staining kit (Roche, Meylan, France) according to the manufacturer's procedure. Fluorescence was measured using the FL2 (AV) and FL3 (propidium iodide, PI) channels of a fluorescence-activated cell sorter apparatus (Calibur cytometer).

### Cell viability

#### XTT

Cells were incubated in a 96-well plate with different effectors for the times indicated in the figure legends. Fifty microliters of sodium 3′-[1-phenylaminocarbonyl)-3,4-tetrazolium]-bis(4-methoxy-6-nitro) benzene sulfonic acid hydrate (XTT) reagent was added to each well. The assay is based on the cleavage of the yellow tetrazolium salt XTT to form an orange formazan dye by metabolically active cells. This bio-reduction occurs in viable cells only and is related to NAD(P)H production through glycolysis. Therefore, the amount of formazan dye measured at 490 nm directly correlated with the number of metabolically active cells reflecting cell proliferation and viability. Each assay was performed in quadruplicate.

#### Cell number - ADAM

For the experiments with C29, we confirmed all the results by evaluating the cell viability. Cell viability and cell death was assessed using the ADAM-MC apparatus (NanoEnTek, Guro-gu, Seoul, Korea) based on fluorescent propidium iodide staining according to the manufacturer's instructions.

### Selectivity index

To determine the selectivity of the activity of the substances tested, the selectivity index (SI) was calculated according to the equation previously described 25 where SI = IC_50_ of a compound in a normal cell line/IC_50_ of the same compound in cancer cell line. The IC_50_ is the concentration required to kill 50% of the cell population.

### Quantitative Real-Time PCR (qPCR) experiments

One microgram of total RNA was used for the reverse transcription, using the QuantiTect Reverse Transcription kit (QIAGEN, Hilden, Germany), with blend of oligo (dT) and random primers to prime first-strand synthesis. SYBR master mix plus (Eurogentec) was used for qPCR. The mRNA level was normalized to 36B4 mRNA. The sequences of oligo nucleotides used in our experiments are describe in **Table S1**.

### *In vitro* stability assay of C29

The *in vitro* stability was determined as followed: 786-O cells were treated with 2.5 µM of compound C29 for the defined time, then lysed with methanol. The lysates were filtered and analyzed by UPLCMS/MS.

### Determination of the pharmacokinetic parameters

The* in vivo* pharmacokinetic parameters were determined in CD-1 mice at a dose of 50 mg/kg after oral administration. The plasma samples (400 µL) were mixed with acetonitrile (1 mL) to precipitate the proteins and extract the compound. After mixing and sonication, proteins were precipitated by centrifugation and the supernatants were analyzed by UPLCMS/MS.

### Tumor xenograft experiments

These studies were carried out in strict accordance with the recommendations in the Guide for the Care and Use of Laboratory Animals. Our experiments were approved by the ''Comité National Institutionnel d'Ethique pour l'Animal de Laboratoire'' (reference: PEA-255 and PEA-277). Cells were injected subcutaneously into the flank of 5-week-old nude (nu/nu) female mice (Janvier). When the tumor reached 100 mm^3^, mice were treated. The tumor volume was determined with a caliper (v = L*l2*0.5).

#### Ectopic model of RCC

***1-***Seven million A498 cells were injected subcutaneously. Mice were treated five times a week for 4 weeks, by gavage with placebo (dextrose water vehicle) or C29 (50 mg/kg).

***2-*** Seven million 786-O cells were injected subcutaneously. Mice were treated trice a week by intraperitoneal injection with placebo (dextrose water vehicle), danirixin (200 µg), C29 (100 µg) or five times a week for four weeks, by gavage with sunitinib (50 mg/kg).

#### Ectopic model of HNSCC

One million CAL33 were injected subcutaneously. Mice were treated five times a week for two weeks, by gavage with placebo (dextrose water vehicle), with danirixin (100 mg/kg) or C29 (100 mg/kg) and once a week by intraperitoneal injection for cisplatin (4 mg/kg).

### Immunohistochemistry (IHC)

Sections of formol-fixed and paraffin-embedded tumors were incubated with monoclonal anti-Ki67 (clone MIB1, DAKO) or anti-CD31 (clone MEC 13.3, BD Pharmingen) antibodies. A biotinylated secondary antibody (DAKO) was applied and binding was detected with the substrate diaminobenzidine against a hematoxylin counterstain.

### Gene expression microarray analysis

Normalized RNA sequencing (RNA-Seq) data produced by The Cancer Genome Atlas (TCGA) were downloaded from cBioportal (www.cbioportal.org, TCGA Provisional; RNA-Seq V2) 26, 27. PFS was defined as the time between surgery and subsequent blood sampling and progression, or death from any cause, censoring live patients and progression free at last follow-up. OS was defined as the time from blood sample collection to the date of death from any cause, censoring those alive at last follow-up. The Kaplan Meier method was used to produce survival curves and significance was assessed using the log-rank test.

### Statistical analysis

All data are expressed as the mean ± the standard error (SD). Statistical significance and p values were determined with the two-tailed Student's *t*-test. One-way ANOVA was used for statistical comparisons. Data were analyzed with Prism 5.0b (GraphPad Software) with a one-way ANOVA with Bonferroni post hoc.

## Results

### The ELR^+^CXCL/CXCR pathway is linked to poor prognosis in RCC and HNSCC

The levels of CXCR1/2 were obtained from the database of patients “The Cancer Genome Atlas (TCGA)”. Of the different tumors, HNSCC and RCC express the highest amounts of CXCR1 (**Figure S2A**). HNSCC is the second and RCC the seventh tumor type expressing the highest amount of CXCR2 (**Figure S2B**). The relevance of targeting the ELR^+^CXCL/CXCR1/2 axis was assessed by correlating the intra-tumor mRNA levels of ELR^+^CXCL cytokines to disease free/progression free (DFS/PFS) (**Table 1A**) and overall survival (OS) (**Table 1B**) of the RCC and HNSCC patients. High levels (> third quartile) of CXCL1-8 except CXCL7 correlated with shorter DFS/PFS and OS for RCC patients whereas only CXCL1 and CXCL3 or CXCL1 and CXCL8 correlated with shorter DFS/PFS and OS, respectively for HNSCC patients (**Table 1A, B**). RCC and HNSCC patients with multiple ELR^+^CXCL mRNA levels above the third quartile had the shortest OS (**Figure S3A-C**). OS was even shorter for RCC and HNSCC patients with ELR^+^CXCL and CXCR1/2 mRNA levels above the third quartile (**Figure S3B-D**). These results strongly suggest that CXCR1/2 may represent a relevant target for the treatment of RCC and HNSCC.

### Selection of C29 as a lead compound

A small focused chemical library of thirty-three new molecules was synthesized and evaluated for anti-proliferative activity against a panel of human tumor cell lines including breast, head and neck, hematologic and kidney tumor cells. The IC_50_ values for each compound were determined and compared to those of SB225002, a CXCR1/2 competitive inhibitor already tested for its effect on the growth of RCC tumor 12, and used as a reference compound; the results are listed in **Table 2**.

*N*, *N'*-diaryureas and thioureas (C1-12) showed limited effect on malignant cells. However, two compounds sharing a common chlorobenzene ring (C9, C12) showed an IC_50_ in the 10 - 20 µmol/L range for solid tumors (breast and kidney), which was higher than the reference molecule SB225002. Other structural changes in the *N*, *N*'-disubstituted ureas, *i.e.,* switching from a phenyl derivative to a benzimidazole or benzothiazole motif, resulted in a higher IC_50_ (compounds C1-12 *vs* C13-33). However, the introduction of a benzoxazole ring (C18, C19) into the compounds did not give anti-proliferation/survival activity. Among the most potent derivatives, compounds C16, C25, C28, C29 and C30, exhibited IC_50_ values below 20 µmol/L for several cancer cell lines. All these hits had a chlorophenyl ring, except compound C30. Among these five hits, only C16 contained an unsubstituted benzimidazole ring, the four other molecules were benzothiazoles substituted at position 6 with a methyl (C25), a nitro (C28, C29) or an ethoxy (C30). Lastly, in the specific case of kidney and head and neck tumor cells, C28 and C29 exerted a dramatic effect on cell proliferation/survival (A498: IC_50_ 5 and 2.5 µmol/L respectively; 786-O: IC_50_ 5 and 2 µmol/L respectively; CAL27: IC_50_ 7 and 2.5 µmol/L respectively; CAL33: IC_50_ 5 and 4 µmol/L respectively), underlying the potency of this class of molecules for treatment of RCC and HNSCC.

Importantly, at a dose of 2.5 µmol/L, C29 did not affect the proliferation/survival of uveal melanoma cells (Mel202), which is consistent with a low level of expression of CXCR1/2 in this tumor type (**Figure S2** and **S4**). Based on this screening, we selected C29 as a lead compound for the following studies. It is worth nothing that C29 features a nitro-benzothiazole moiety that has not been reported before, attesting to the originality of this new class of bioactive compounds.

### C29 docking to CXCR1

**Figure 2:**
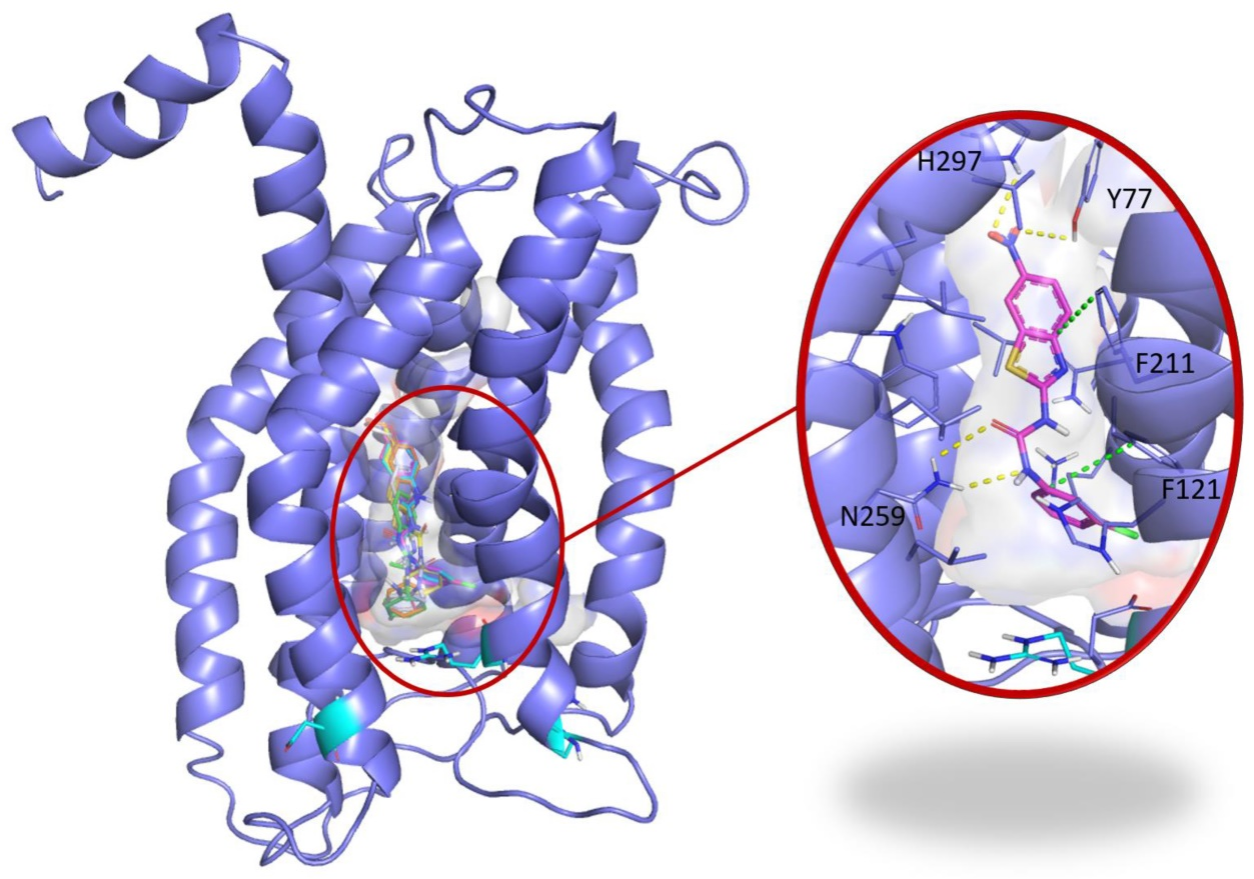
**Blind docking study of C29 in CXCR1**. Blind docking studies of the CXCR1 structure. Left panel: C13 (green), C14 (cyan), C26 (yellow), C28 (orange) and C29 (magenta) best scored poses and C30 (white) second best scored pose are all located in a central buried pocket of the CXCR1 structure. Right panel: detailed view of the predicted interactions between C29 and CXCR1 residues with hydrogen bonds in yellow dashed lines and pi-stacking in green dashed lines (distances C29-H297: 2.8 Å, C29-Y77: 3.1 Å, C29(CO)-N259: 2.9 Å, C29(NH)-N259: 2.9 Å). The CXCR1 residues surrounding the central buried pocket are visualized as a stick representation. The 3 CXCR1 residues R199, R203 and D265, previously identified by Alanine scanning experiments to be involved in the CXCR1/CXCL8 interaction are represented as cyan sticks in both panels.

To address the specificity of C29 for CXCR1/2, we performed a blind docking approach. Several areas of CXCR1 were identified as possible C29 binding sites. However, we noted that 14 out of the 15 top poses ranked according to their AutoDock Vina scores, i.e. the 5% poses with the best scores, were all located at the same binding site. This binding site is a central buried pocket, the volume of which varies among the ten available conformations of CXCR1. The cytokine binding site contains Arg203, a residue previously identified by Alanine scanning experiments to be involved in the CXCR1/CXCL8 interaction 28. We thus propose a binding model, in which C29 is located in a buried central pocket of CXCR1 close to Arg203 (**Figure 2**). This finding supports the hypothesis that C29-CXCR1 binding will impede CXCL8 interaction with CXCR1.

### C29 inhibited ELR+CXCL-mediated proliferation and migration of endothelial cells

**Figure 3:**
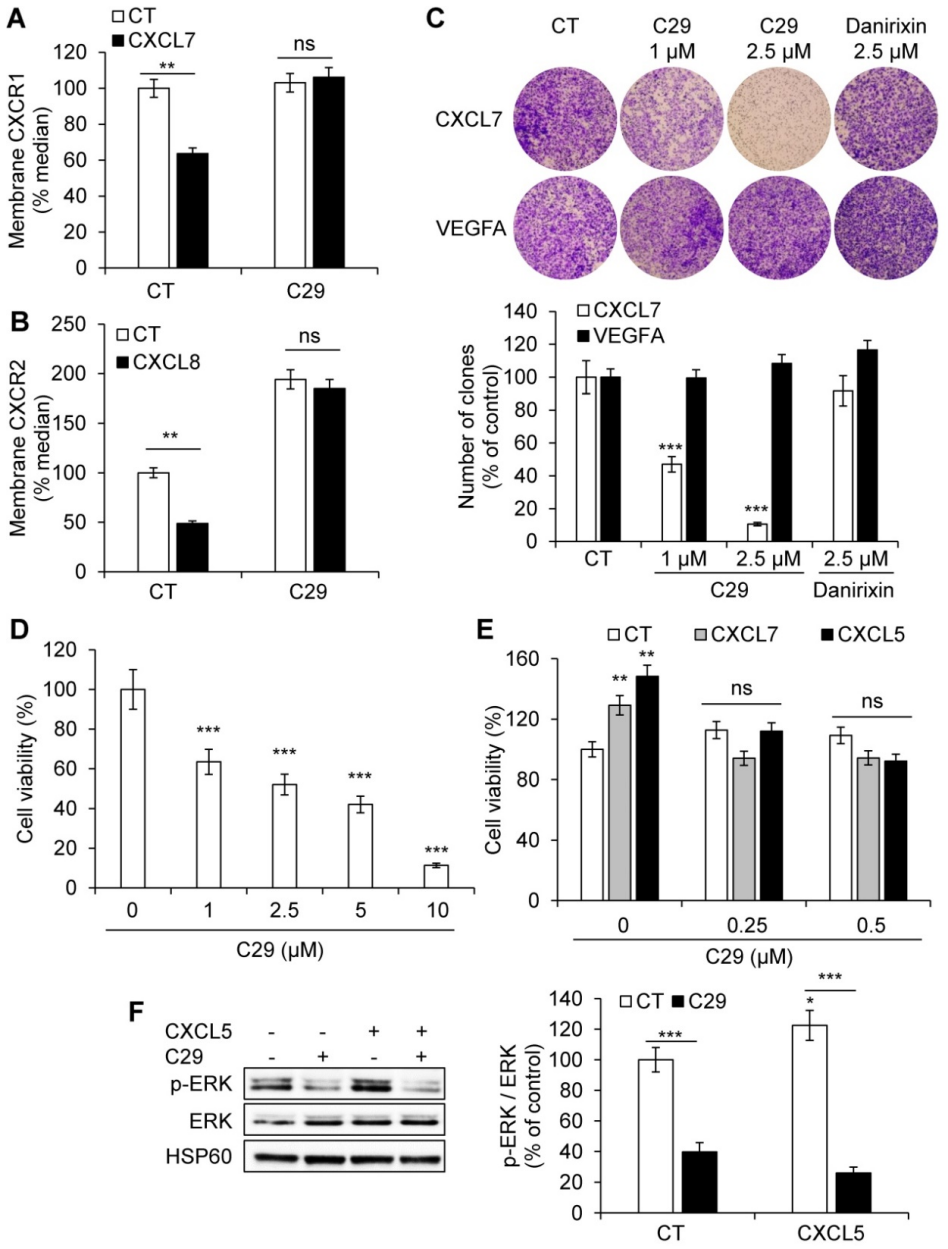
** C29 inhibited the ERL+CXCL/CXCR1/2 axis in endothelial cells**. (**A**) HuVEC were stimulated with 25 ng/ml CXCL7 during 1 h. Membrane-associated CXCR1 protein levels were quantified by flow cytometry. (**B**) HuVEC were stimulated with 25 ng/ml CXCL8 during 1 h. Membrane-associated CXCR2 protein levels were quantified by flow cytometry. (**C**) CXCL7 (50 ng/ml) or VEGFA (50 ng/ml)-dependent HuVEC migration was analyzed using Boyden chamber assays in the presence/absence of C29 (1 or 2.5 µM) or danirixin (2.5 µM). (**D**) HuVEC were grown in the presence of different concentrations of C29 for 48 h. Cell viability was measured with the XTT assay. (**E**) HuVEC were incubated with 100 ng/ml CXCL5 or CXCL7, in the presence of the indicated concentrations of C29 for 48 h. Cell viability was measured with the XTT assay. (**F**) HuVEC were pre-treated with 5 µM C29 for 1 h then stimulated with 50 ng/ml CXCL5 for 10 min. p-ERK levels were analyzed by immunoblotting. ERK and HSP60 served as loading controls. Results are represented as the mean of three independent experiments ± SEM. Statistical significance was determined using an unpaired Student's *t-*test: *P<0.05; **P<0.01; ***P<0.001.

CXCR1/2 are expressed on endothelial cells and participate in ELR^+^CXCL-mediated angiogenesis. We assessed the ability of C29 to inhibit CXCR1/2 in normal endothelial cells (HuVECs). CXCR1/2 are internalized upon stimulation, a process mainly involved in signal desensitization associated with G protein coupled receptors and resulting in the transfer of receptors from the plasma membrane to the endosomal compartment 29. Hence, modification of plasma membrane levels reflects, in part, stimulation. Following CXCL7 or CXCL8 stimulation in the presence of C29 (2.5 µmol/L), the CXCR1 or CXCR2 levels at the plasma membrane were increased suggesting that C29 prevented CXCL7/8-dependent internalization of their receptors (**Figure 3A-B**).

C29 decreased CXCL7-dependent but not VEGFA-dependent migration of HuVECs at the same concentration (2.5 µmol/L). Danirixin, a competitive inhibitor of CXCR2 and to a lesser extent of CXCR1 30, tested in phase II clinical trials for the treatment of Respiratory Syncytial Virus (RSV) infections, had no effect at this concentration (**Figure 3C**,** Figure S5**). This result suggests stronger efficacy of C29 compared to danirixin. C29 also inhibited basal and CXCL5/CXCL7-dependent HuVECs viability (**Figure 3 D-E**), which was consistent with inhibition of the activity of basal and CXCL5-dependent ERK/MAP Kinase signaling pathway (**Figure 3F**). Hence, C29 has a strong inhibitory effect on an alternative angiogenic pathway that compensates for the inhibition of the VEGF/VEGFR axis 31.

### C29 is equally efficient on naive and resistant RCC and HNSCC cells

**Figure 4:**
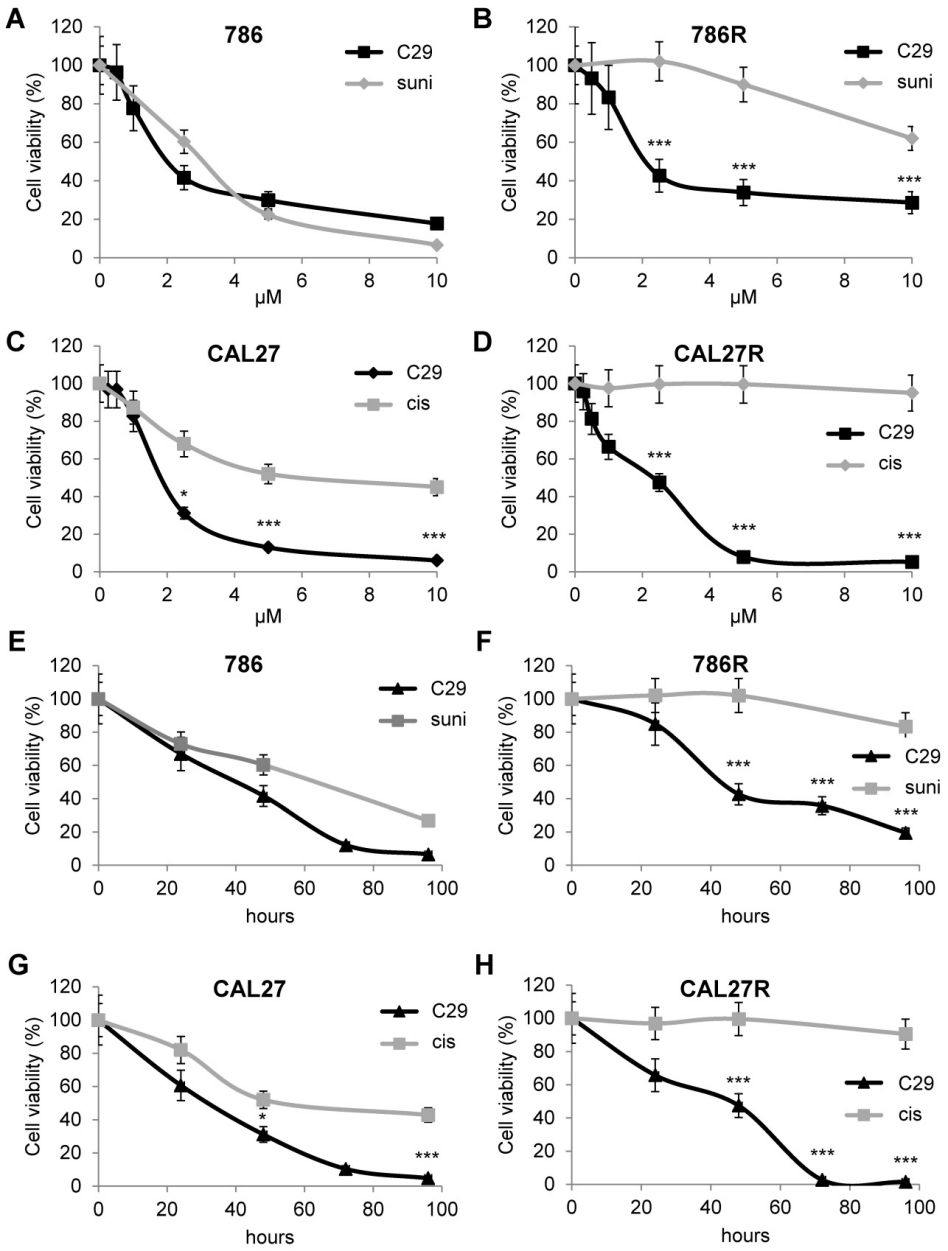
** C29 decreased the viability of sensitive and resistant RCC and HNSCC cells.** (**A**) and (**B**) RCC naive (786, **A**), and sunitinib-resistant (786R, **B**) 786-O cells, were treated with C29 or sunitinib (1 to 10 µM) for 48 h. Cell viability was measured with the XTT assay. (**C**) and (**D**), HNSCC naive (CAL27, **C**), and cisplatin-resistant (CAL27R, **D**) CAL27 cells, were treated with C29 or cisplatin (1 to 10 µM) for 48 h. Cell viability was measured with XTT assays. (**E**) and (**F**), 786 (**E**), 786R (**F**), were treated with 2.5 µM C29 or 2.5 µM sunitinib for 24 to 96 h. Cell viability was measured with the XTT assay. (**G**) and (**H**), CAL27 (**G**), CAL27R (**H**), were treated with 2.5 µM C29 or 2.5 µM cisplatin for 24 h to 96 h. Cell viability was measured with the XTT assay. Results are represented as the mean of three independent experiments ± SEM. Statistical significance was determined using an unpaired Student's *t-*test: *P<0.05; **P<0.01; ***P<0.001.

**Figure 5:**
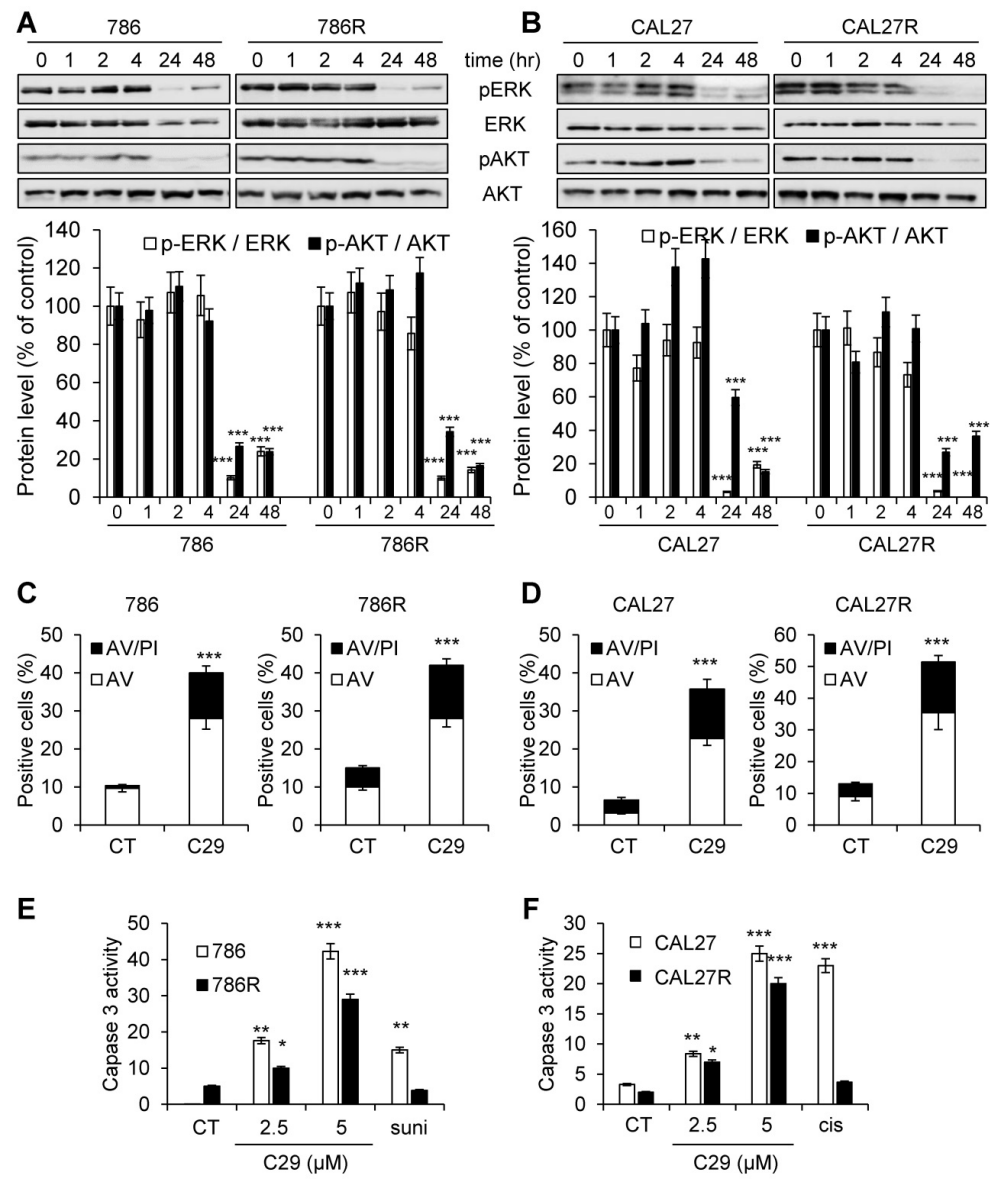
** C29 inhibited signaling pathways associated with cell proliferation and survival, and induced apoptotic cell death of sensitive and resistant RCC and HNSCC cells.** (**A**) and (**B**), 786 and 786R cells (**C**), and CAL27 and CAL27R (**D**) were treated with 2.5 µM C29 for 1 h to 48 h. p-ERK and p-AKT levels were determined by immunoblotting. ERK and AKT served as loading controls. (**C**) and (**D**), 786 and 786R cells (**C**), and CAL27 and CAL27R (**D**) were treated with 2.5µM C29 for 48 h. Histograms show both annexin-V^+^/PI^-^ cells (white bars) and annexin-V^+^/PI^+^ cells (black bars). (**E**) and (**F**), 786 and 786R cells (**E**), and CAL27 and CAL27R (**F**) were treated with 2.5 and 5 µM C29, or with 2.5 µM sunitinib (suni) or with 3 µM cisplatin (cis) for 48 h. The caspase 3 activity was evaluated using 0.2 mM Ac-DEVD-AMC as substrate. Results expressed as arbitrary units (A.U.) are means ± standard deviation of three independent experiments. Statistical significance was determined using an unpaired Student's *t-*test: *P<0.05; **P<0.01; ***P<0.001.

The first-line treatments for RCC and HNSCC are respectively, sunitinib and cisplatin but relapse is ineluctably observed. Hence, our objectives were to; i) compare the efficacy of reference treatments to that of C29; ii) evaluate if C29 may be relevant at relapse as current standard of care. Dose responses and time courses were performed on naive cells and on sunitinib 32 and cisplatin-resistant cells to compare the C29 efficiency. C29 inhibited the viability of sensitive 786-O RCC cells as efficiently as sunitinib (786, **Figure 4A-E**,** Figure S6A**). The effect of C29 on cell viability was conserved in sunitinib-resistant 786-O cells (786R, **Figure 4B-F**,** Figure S6A**). The effect of C29 on CAL27 HNSCC cell viability was greater than that of cisplatin (**Figure 4C-G**,** Figure S6B**), and the effect was equivalent for cisplatin-resistant CAL27 cells (CAL27R, **Figure 4D-H**, **Figure S6B**). Compared to C29, the reference CXCR1/2 inhibitor SB225002 had a modest effect on RCC and HNSCC cells (**Figure S7**). C29 inhibited the ERK/MAP Kinase and PI3K/AKT signaling pathways, two major pathways involved in cell proliferation, of naive and resistant RCC and HNSCC cells (**Figure 5A-B**). This result suggests that ELR^+^CXCL cytokines produced by RCC 31 and HNSCC 33 cells stimulate a CXCR1/2-dependent autocrine proliferation loop. C29 induced cell death in sensitive and resistant cells (**Figure 5C-D**). The apoptotic cell death induced by C29 was confirmed by an increase in the caspase 3 activity in both cell types (**Figure 5E-F**).

To further explore the effect of C29 we compared its activity on tumor kidney cells to that on primary normal cells 34. C29 significantly decreased the proliferation of primary kidney tumor cells (CC, TF, **Figure S8A-B**) but had no effect on primary normal kidney cells (15S), even when C29 was used at a higher concentration (5 µmol/L). FACS analysis detected apoptotic markers in TF and CC cells in the presence of C29 at 1 µmol/L, which was not the case for normal cells (15S, **Figure S8C**). The results with primary cells were similar to those described above for kidney cell lines.

We then calculated the selectivity index (SI) to evaluate the selectivity to the targets. The normal primary kidney cells (15S) served as the reference value. The SI was superior to that of Mel202 for all tumor cells (**Table 3**), which is in favor of a specific effect on CXCR1/2 receptors.

### C29 inhibited the growth of experimental RCC and HNSCC

**Figure 6:**
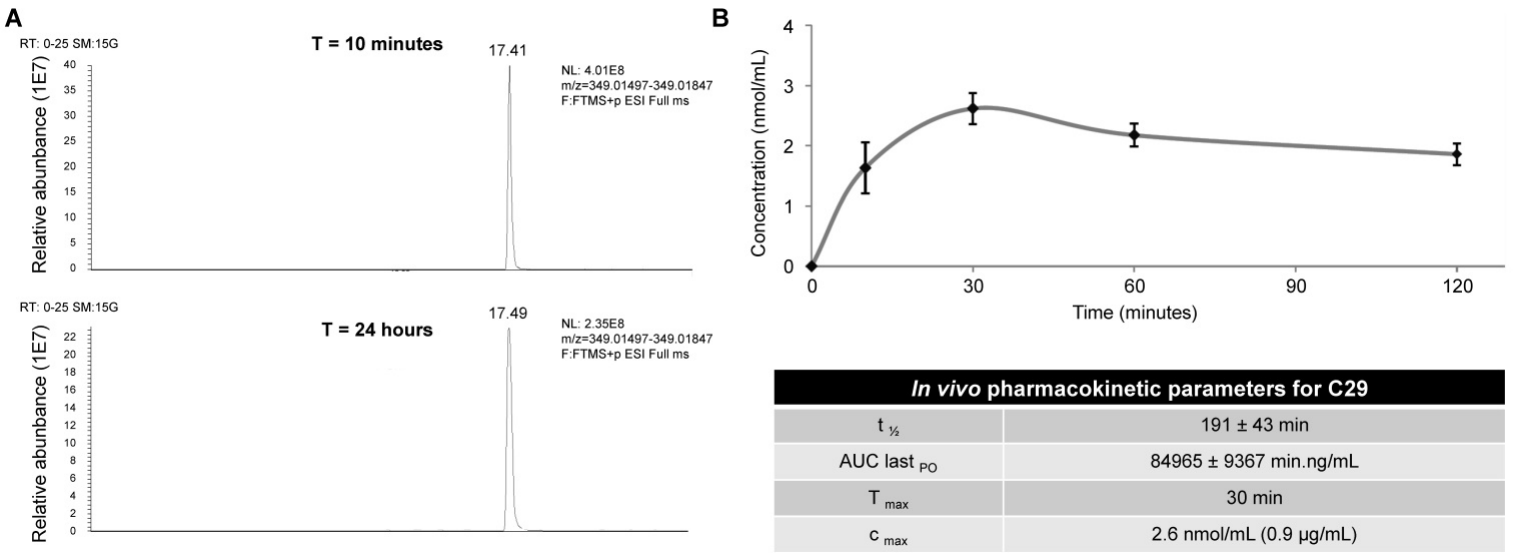
** Determination of the *in cellulo* (A) stability and *in vivo* pharmacokinetic parameters (B) of compound C29. (A)** 786-O cells were treated with 2.5 µM of C29 for 24 h at room temperature, then cells were lyzed with methanol. UPLCMS/MS analyses highlighted the absence of degradation of the compound at this time point. **(B)** C29 was formulated at 7.6 mg/mL and administrated by oral gavage at 50 mg/kg in CD-1 mice. The plasma samples (400 µL) were mixed with acetonitrile (1 mL) and sonicated. Proteins were precipitated by centrifugation and the supernatants were analyzed by UPLCMS/MS.

To be confident about using C29 for *in vivo* experiments in mice, we first tested its stability by performing UPLC/HRMS analyzes on the 786-O cells. No degradation of C29 was observed after a 24 h-treatment at room temperature (**Figure 6A**), attesting to its high stability *in cellulo.* C29 was then formulated at 7.6 mg/mL and administrated by oral gavage at 50 mg/kg. C29 exhibited a half-life of 190 min, combined with a C_MAX_ of 0.9 µg/ml at 30 min (**Figure 6B**). Global exposure remained high and the AUC was close to 85000 min.ng/m. These results prompted us to evaluate C29 on the growth of experimental tumors in mice.

**Figure 7:**
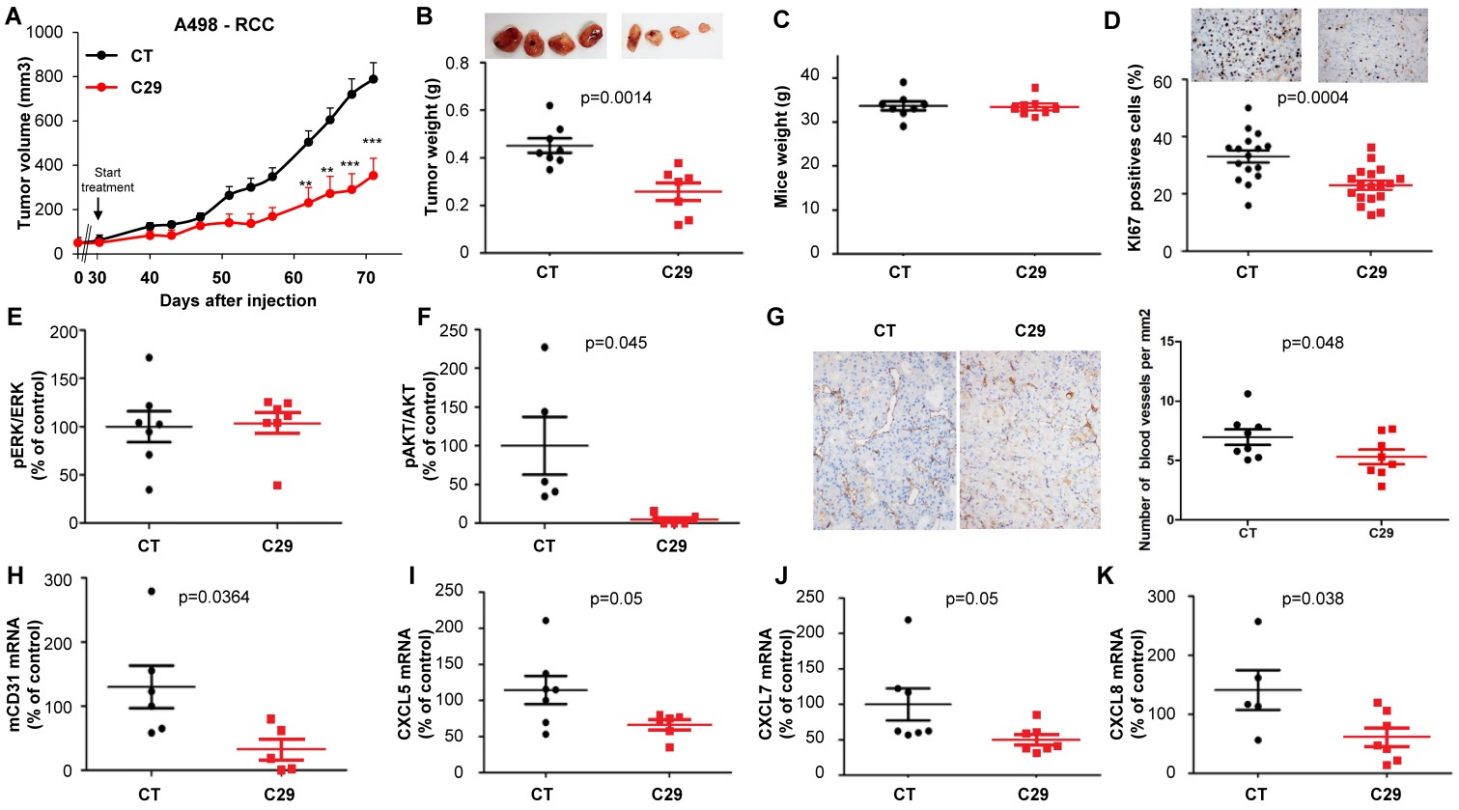
***In vivo* mouse RCC xenograft experiments.** Seven million A498 cells were injected subcutaneously into the flank of nude mice. When the tumor reached 100 mm^3^, mice were treated five times a week for four weeks by gavage with placebo (dextrose water vehicle) or C29 (50 mg/kg). (**A**) The tumor volume was measured twice weekly. The results are presented as the means ± sd. (**B**) Tumor weights at the end of the experiment. (**C**) Weights of the animals at the end of the experiment. (**D**) Human Ki67 expression in untreated and treated mice. The number of proliferative cells was determined by calculating the ratio of colocalized 4,6 diamidino-2-phenylindole (DAPI)/Ki67-positive cells with respect to the total cell number. (**E**) and (**F**) The levels of pERK, ERK, pAKT and AKT in tumor lysates were determined by immunoblotting. The graphs represent the ratio of pERK (**E**) or pAKT (**F**) to non-phosphorylated ERK or AKT. (**G**) Blood vessels were visualized by IHC for CD31. (**H-K**) The levels of human VEGFA, CXCL5, CXCL7 and CXCL8 mRNA in tumors were evaluated by qPCR. Statistical significance was determined using an unpaired Student's *t-*test: *P<0.05; **P<0.01; ***P<0.001.

**Figure 8:**
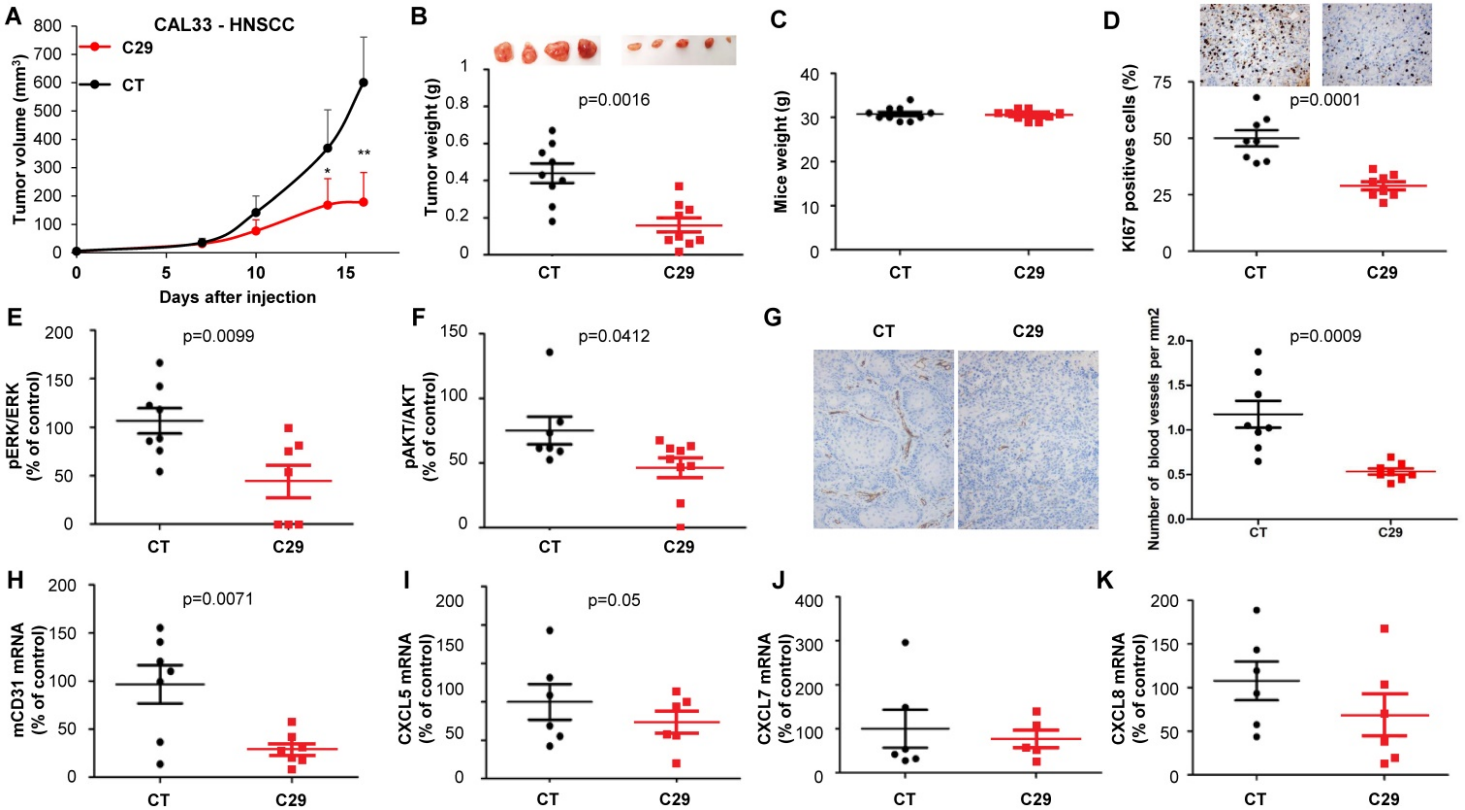
***In vivo* mouse HNSCC xenograft experiments.** One million CAL33 cells were injected subcutaneously into the flank of nude mice. When the tumor reached 100 mm^3^, mice were treated five times a week for two weeks by gavage with placebo (dextrose water vehicle) or C29 (100 mg/kg). (**A**) The tumor volumes are presented as the means ± sd. (**B**) Tumor weights at the end of the experiment. (**C**) Weights of the animals at the end of the experiment. (**D**) Human Ki67 expression in untreated and treated mice. The number of proliferative cells was determined by calculating the ratio of colocalized 4,6 diamidino-2-phenylindole (DAPI)/Ki67-positive cells with respect to the total cell number. (**E**) and (**F**) The levels of pERK, ERK, pAKT and AKT in tumor lysates were determined by immunoblotting. The graphs represent the ratio of pERK (**E**) or pAKT (**F**) to non-phosphorylated ERK or AKT. (**G**) Blood vessels were visualized by IHC for CD31. (**H-K**) The level of human VEGFA, CXCL5, CXCL7 and CXCL8 mRNA in tumors were evaluated by qPCR. Statistical significance was determined using an unpaired Student's *t-*test: *P<0.05; **P<0.01; ***P<0.001.

C29 slowed-down the growth of experimental RCC or HNSCC with a decrease of more than 65% of the tumor volume at the end of the experiment (**Figure 7A-8A**). This result correlated with the decrease in the tumor weight of 40 or 65% for RCC and HNSCC, respectively (**Figure 7B-8B**). Moreover, we did not observe any weight loss of the animals in the treated group, which suggests that this molecule did not exert acute toxicity (**Figure 7C-8C**). C29 decreased the proliferation marker Ki-67 (**Figure 7D-8D**) and inhibited PI3K/AKT but not the ERK pathway in experimental RCC (**Figure 7E-F**) and both pathways in experimental HNSCC (**Fig. 8E-F**). The mRNA level of murine CD31, a relevant marker of blood vessels, was decreased for both tumor types (**Figure 7G-8G**). The mRNA levels of ERL^+^CXCL cytokines (CXCL5/7/8), but not of VEGFA, were significantly decreased by C29 in experimental RCC (**Figure 7H-K**)**.** Only CXCL5 was decreased in experimental HNSCC (**Figure 8H-K**)**.** These results were consistent with the decrease in blood vessels visualized by the down-regulation of CD31 levels on IHC (**Figure 7G- 8G**).

The concentration in blood (C_MAX_) of C29 once administered by oral gavage was 2.6 µmol/L (comparable to the *in vitro* IC_50_), which means that a small amount of the drug crossed the gastric barrier. Therefore, we tested the efficacy of C29 administered by intra-peritoneal injection. Three injections per week of 100 µg C29 was as efficient as a dose of 50 mg/kg administered by oral gavage (**Figure S9A-B**). An equivalent efficacy was obtained with danirixin at 200 µg three times a week or sunitinib 50 mg/kg by oral gavage five times a week (**Figure S9C-D**). C29 administered by oral gavage at a dose of 100 mg/kg five times a week was more efficient than danirixin, at the same dose, or than the reference treatment with cisplatin on experimental HNSCC (**Figure S9E-F**). C29 treatment (100mg/kg by oral gavage five times a week for three weeks) did not induce hematological, renal or hepatic toxicity (**Figure S10A-D**). These results strongly suggest that C29 may represent a relevant therapeutic tool for RCC and HNSCC.

## Discussion

The therapeutic options for RCC and HNSCC at a metastatic stage are anti-angiogenic drugs or immunotherapy, alone or in combination 35, 36, and a combination of radio/chemotherapy, respectively 37. Despite an increase in time to progression, the patients ineluctably relapse within a few months 38. We have already demonstrated that the CXCL/CXCR axis is involved in relapse of RCC patients 12, 33. We report herein the identification and biological characterization of a new series of CXCR1/2 inhibitors with significant anti-cancer effects. The lead compound of this series, C29, inhibited tumor cell viability and angiogenesis, two major hallmarks of aggressive cancers 39. This study highlighted the possibility of targeting CXCL/CXCR crosstalk using a potent CXCR1/2 inhibitor, as a relevant therapeutic option to treat incurable RCC and HNSCC. C29 showed higher efficacy in two independent tumor types as compared to the older generation of competitive inhibitors SB225002 or danirixin.

Targeting the CXCL/CXCR signaling pathway induced; i) inhibition of proliferation of tumor and endothelial cells, ii) tumor cell death, and iii) inhibition of tumor vascularization. A high expression level of ELR^+^CXCL cytokines and their receptors in RCC and HNSCC suggested that CXCR inhibitors may be considered as relevant first-line treatment. Since C29 may represent an option on relapse when on reference therapies early phase clinical trials can be rapidly performed. The rationale in the case of therapeutic failure is sound for both tumors.

We observed that ELR^+^CXCL cytokines were induced in response to the reference anti-angiogenic treatment sunitinib in RCC 24 and in response to radiotherapy in HNSCC 26. These results represent a relevant rationale to administer CXCR inhibitors on relapse.

RCC and HNSCC are inflamed tumors and therefore eligible for immunotherapy. Indeed, immunotherapy improved the outcome of RCC 35, 40 and HNSCC 41. However, only 30% of patients benefit from these treatments 35, 42. The presence of myeloid-derived suppressor cell (MDSC) partly explains the limited effect of immunotherapies in some patients 31, 33. A correlation between the intra-tumor expression of CXCL5, CXCL8 and IL1β, and the presence of MDSC, creating an immuno-suppressive environment, has been recently highlighted in RCC 43. Moreover, we demonstrated the IL1β controls the expression of CXCL7, one of the main CXCL cytokines involved in RCC aggressiveness 12. Thus, by decreasing CXCR-expressing MDSC, C29 might reactivate the anti-tumor immune response. In addition to its anti-viability and anti-angiogenic effects, C29 may serve indirectly as an immune check point inhibitor and can also sensitize tumors to immunotherapy by decreasing the level of cytokines favoring anergia. Therefore, C29 has the ability to target four hallmarks of cancers; proliferation of tumor cells, tumor angiogenesis, chronic inflammation and immune-tolerance. Our efforts are currently directed towards the validation of this hypothesis, and the results will be reported in due course.

## Supplementary Material

Supplementary figures.

## Figures and Tables

**Table 1:**
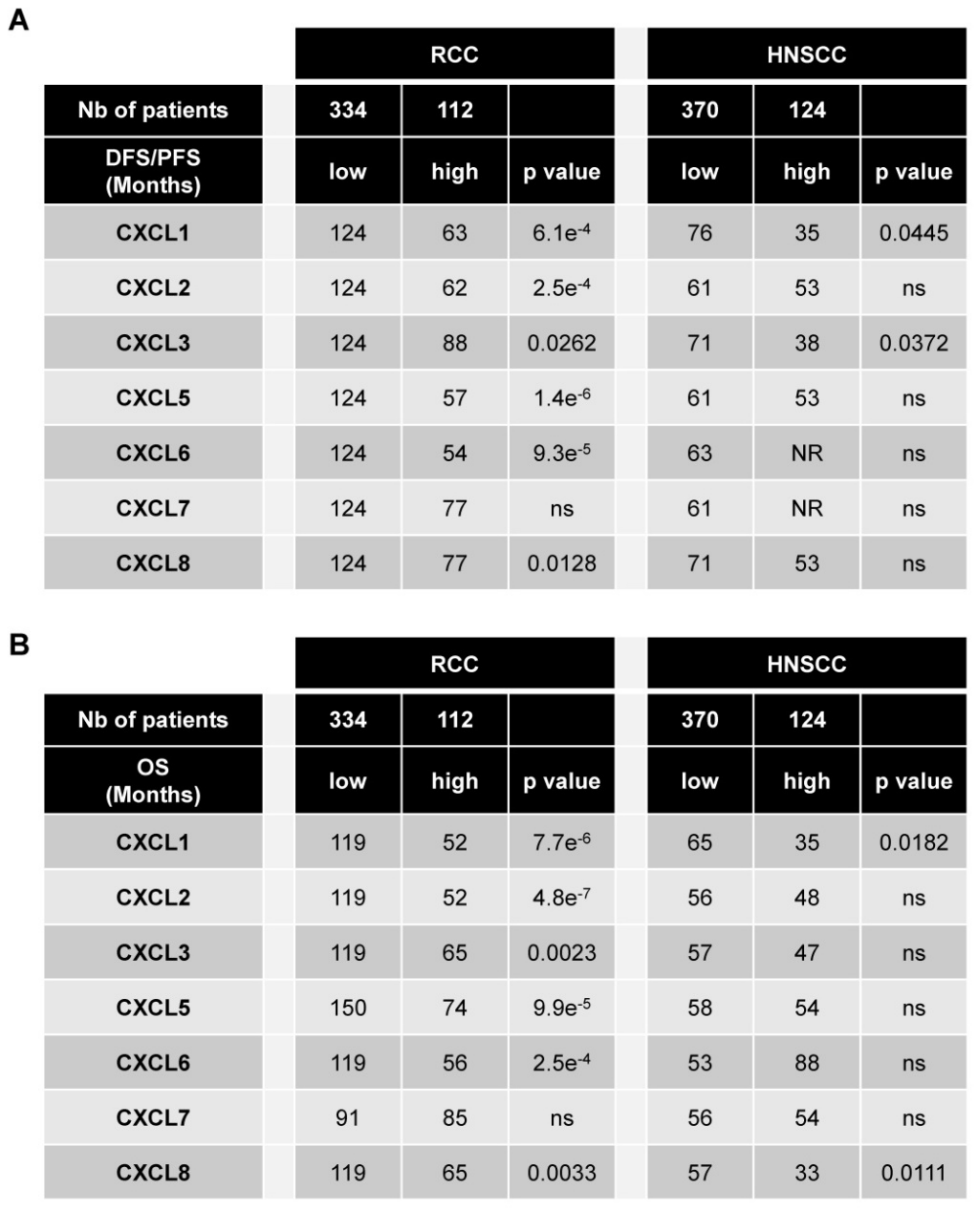
** CXCL cytokines are associated with poor prognosis in RCC and in HSNCC.** 446 RCC and 494 HNSCC patients were analyzed using the “The Cancer Genome Atlas (TCGA)”. The levels of CXCL1/2/3/5/6/7/8 mRNA correlated with disease/progression free survival (DSF/PFS) or with overall survival (OS). The third quartile value of CXCL expression was chosen as the reference. Statistical significance (*p* values) is indicated.

**Table 2:**
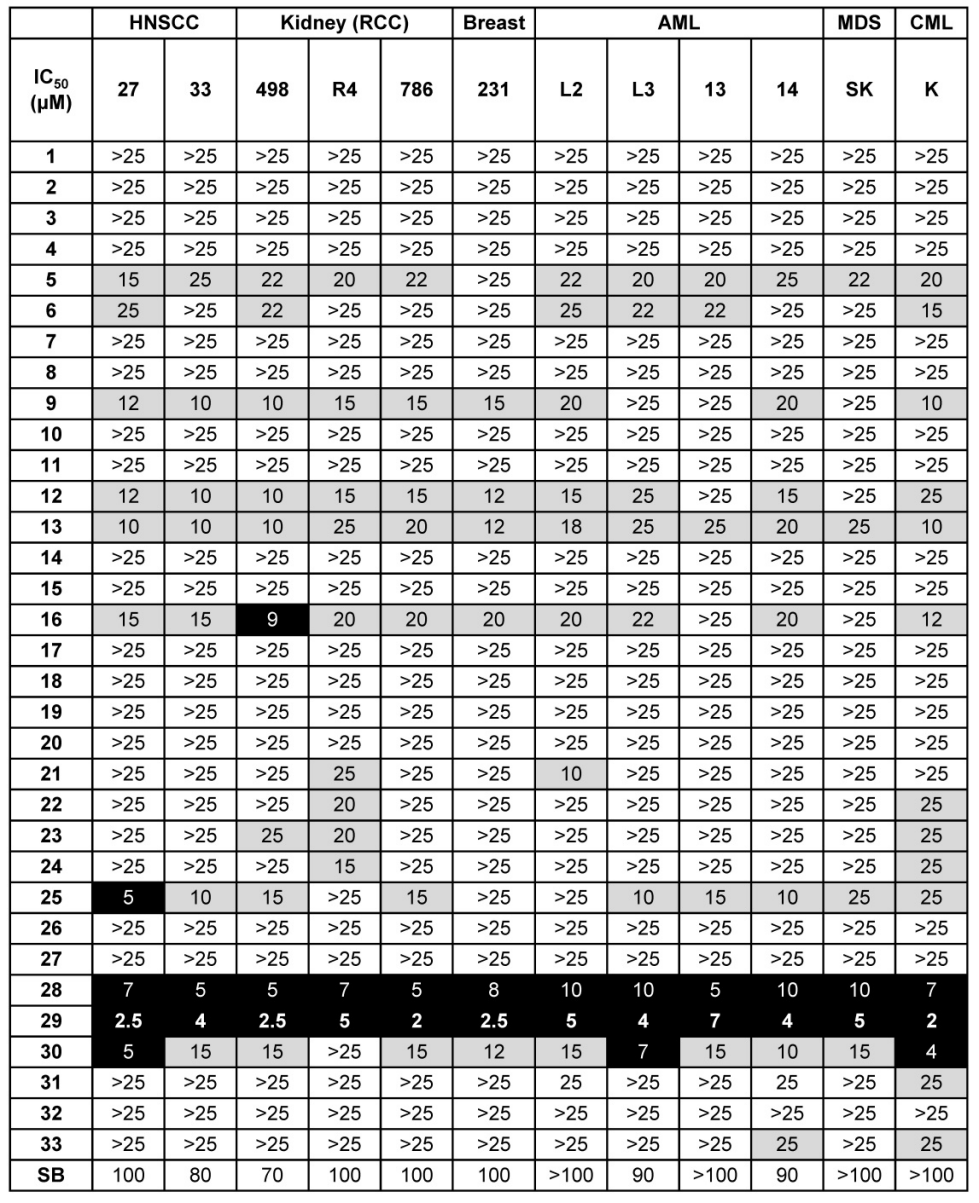
** Screening of the 33 newly synthesized *N, N'*-diarylureas and thioureas using different solid and hematological tumor cell lines.** Values are reported as IC_50_ measured with the XTT assay (48 h). The results are expressed in μM, and all the IC_50_ values given in this table showed a standard deviation of 10%. Results are represented as the mean of three independent experiments.

**Table 3:**
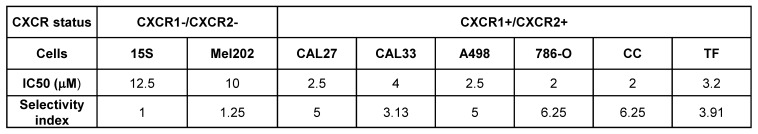
Comparison of IC_50_ and the selectivity index for the different cell lines

## Abbreviations

DFS: disease-free survival
HNSCC: Head and Neck Squamous Cell Carcinoma
HuVEC: human endothelial cells
IHC: immunohistochemistry
MDSC: myeloid-derived suppressor cells
OS: overall survival
PFS: progression-free survival
RCC: clear cell renal cell carcinoma
TCGA: The Cancer Genome Atlas
