# Supplementary material for: New CXCR1/CXCR2 inhibitors represent an effective treatment for kidney or head and neck cancers sensitive or refractory to reference treatments
Source: Theranostics. 2019 Jul 9;9(18):5332–46. doi: 10.7150/thno.34681 (PMC6691587; doi:10.7150/thno.34681)
Supplement: Supplementary file 1 — Supplementary figures. [file thnov09p5332s1.pdf]

N°	Structure	IUPAC name	Molecular Weight (g/mol)	HPLC purity (λ280)	NMR spectrums	HRMS
1	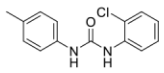	1-(2-chlorophenyl)-3-(*p* -tolyl)urea	260,72	99.7% (λ280)	**^{1}H NMR** (200 MHz, DMSO-*d6*): δ 9.32 (s, 1H, N-H), 8.27 (s, 1H, N-H), 8.17 (d, *J* = 8.1 Hz, 1H, H_{Ar}), 7.50 – 7.20 (m, 4H, H_{Ar}), 7.18 – 6.94 (m, 3H, H_{Ar}), 2.25 (s, 3H, CH_3); **^{13}C NMR** (50 MHz, DMSO-*d6*): δ 152.14, 136.87, 136.07, 130.97, 129.29 (2C), 129.20, 127.57, 123.15, 121.76, 121.19, 118.27 (2C), 20.37.	$[M+H]^+$ calc. for $C_{14}H_{14}ClN_2O^+$, 261.08, found **261.80**
2	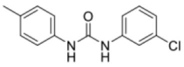	1-(2-chlorophenyl)-3-(2,4-dichlorophenyl)urea	260,72	93,90%	**^{1}H NMR** (200 MHz, DMSO-*d6*): δ 8.84 (s, 1H, N-H), 8.64 (s, 1H, N-H), 7.70 (s, 1H, H_{Ar}), 7.30 (dd, *J* = 11.0, 8.0 Hz, 4H, H_{Ar}), 7.09 (d, *J* = 8.2 Hz, 2H, H_{Ar}), 7.00 (dd, *J* = 7.0, 1.3 Hz, 1H, H_{Ar}), 2.24 (s, 3H, CH_3); **^{13}C NMR** (50 MHz, DMSO-*d6*): δ 152.41, 141.39, 136.82, 133.19, 130.94, 130.37, 129.20 (2C), 121.30, 118.49 (2C), 117.46, 116.55, 20.35.	$[M+H]^+$ calc. for $C_{14}H_{14}ClN_2O^+$, 261.08, found **261.80**
3	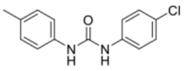	1-(4-chlorophenyl)-3-(*p* -tolyl)urea	260,72	98,50%	**^{1}H NMR** (200 MHz, DMSO-*d6*): δ 8.76 (s, 1H, N-H), 8.59 (s, 1H, N-H), 7.47 (d, *J* = 8.4 Hz, 2H, H_{Ar}), 7.32 (d, *J* = 5.9 Hz, 4H, H_{Ar}), 7.08 (d, *J* = 8.0 Hz, 2H, H_{Ar}), 2.23 (s, 3H, CH_3); **^{13}C NMR** (50 MHz, DMSO-*d6*): δ 152.47, 138.84, 136.94, 130.82, 129.20 (2C), 128.61 (2C), 125.19, 119.63 (2C), 118.41 (2C), 20.36.	$[M+H]^+$ calc. for $C_{14}H_{14}ClN_2O^+$, 261.08, found **261.80**
4	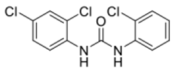	*1* -(2-chlorophenyl)-3-(2,4-dichlorophenyl)urea	315,58	96,30%	**^{1}H NMR** (200 MHz, DMSO-*d6*): δ 9.27 – 8.92 (m, 2H, N-H), 8.09 (t, *J* = 10.2 Hz, 2H, H_{Ar}), 7.63 (s, 1H, H_{Ar}), 7.51 – 7.24 (m, 3H, H_{Ar}), 7.06 (t, *J* = 7.3 Hz, 1H, H_{Ar}); **^{13}C NMR** (50 MHz, DMSO-*d6*): δ 152.12, 135.58, 135.03, 129.34, 128.66, 127.57, 127.51, 126.62, 124.00, 123.35, 123.16, 122.86, 122.54	$[M+H]^+$, calc. for $C_{13}H_{10}Cl_3N_2O^+$, 314.98, found **314.87**
5	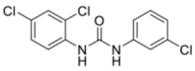	1-(3-chlorophenyl)-3-(2,4-dichlorophenyl)urea	315,58	98,00%	**^{1}H NMR** (200 MHz, DMSO-*d6*): δ 9.63 (s, 1H, N-H), 8.45 (s, 1H, N-H), 8.17 (d, *J* = 9.0 Hz, 1H, H_{Ar}), 7.73 (s, 1H, H_{Ar}), 7.63 (d, *J* = 1.9 Hz, 1H, H_{Ar}), 7.44 – 7.19 (m, 3H, H_{Ar}), 7.05 (d, *J* = 7.6 Hz, 1H, H_{Ar}); **^{13}C NMR** (50 MHz, DMSO-*d6*): δ 151.89, 140.79, 134.94, 133.31, 130.54, 128.60, 127.67, 126.44, 122.91, 122.33, 121.92, 117.62, 116.68.	$[M+H]^+$, calc. for $C_{13}H_{10}Cl_3N_2O^+$, 314.98, found **314.13**
6	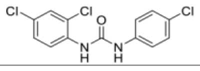	*1* -(4-chlorophenyl)-3-(2,4-dichlorophenyl)urea	315,58	98,40%	**^{1}H NMR** (200 MHz, DMSO-*d6*): δ 7.51-7.32 (m, 4H, H_{Ar}), 7.62 (s, 1H, H_{Ar}), 8.18 (d, *J* = 8.0 Hz, 2H, H_{Ar}), 8.42(s, 1H, N-H), 9.57 (s, 1H, N-H); **^{13}C NMR** (50 MHz, DMSO-*d6*): δ 151.91, 138.26, 135.06, 128.77 (2C), 128.58, 127.66, 126.28, 125.80, 122.78, 122.20, 119.76 (2C).	$[M+H]^+$, calc. for $C_{13}H_{10}Cl_3N_2O^+$, 314.98, found **314.87**
7	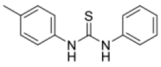	1-phenyl-3-(*p* -tolyl)thiourea	242,34	95,70%	**^{1}H NMR** (200 MHz, DMSO-*d6*): δ 9.72 (s, 2H, 2N-H), 7.45 (d, *J* = 7.6 Hz, 2H, H_{Ar}), 7.29 (t, *J* = 7.8 Hz, 4H, H_{Ar}), 7.16 – 7.02 (m, 3H, H_{Ar}), 2.24 (s, 3H, CH_3); **^{13}C NMR** (50 MHz, DMSO-*d6*); δ 179.57, 139.54, 136.81, 133.67, 128.91 (2C), 128.40 (2C), 124.31, 123.83 (2C), 123.58 (2C), 20.55.	$[M+H]^+$, calc. for $C_{14}H_{15}N_2S^+$, 243.09, found **243.13**
8	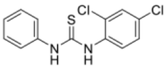	1-(2,4-dichlorophenyl)-3-phenylthiourea	297,2	98,60%	**^{1}H NMR** (200 MHz, DMSO-*d6*): δ 10.08 (s, 1H, N-H), 9.48 (s, 1H, N-H), 7.68 (d, *J* = 2.2 Hz, 1H, H_{Ar}), 7.62 (d, *J* = 8.6 Hz, 1H, H_{Ar}), 7.42 (ddd, *J* = 15.8, 15.3, 7.5 Hz, 5H, H_{Ar}), 7.16 (t, *J* = 7.1 Hz, 1H, H_{Ar}); **^{13}C NMR** (50 MHz, DMSO-*d6*): δ 180.37, 139.06, 135.77, 131.27, 131.04, 130.93, 128.94, 128.66 (2C), 127.39, 124.95, 123.93 (2C)	$[M+H]^+$ calc. for $C_{13}H_{11}Cl_2N_2S^+$, 297.00, found **297.13**
9	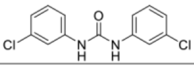	1,3-bis(3-chlorophenyl)urea	281,14	98,80%	**^{1}H NMR** (400 MHz, DMSO-*d6*): δ 8.96 (s, 2H, N-H), 7.70 (t, *J* = 1.7 Hz, 2H, H_{Ar}), 7.34 – 7.24 (m, 4H, H_{Ar}), 7.03 (dt, *J* = 7.3, 1.8 Hz, 2H, H_{Ar}); **^{13}C NMR** (101 MHz, DMSO-*d6*): δ 152.25, 140.99 (2C), 133.20 (2C), 130.41 (2C), 121.71 (2C), 117.76 (2C), 116.84 (2C).	$[M+H]^+$ calc. for $C_{13}H_{11}Cl_2N_2S^+$, 281.02, found **281.13**
10	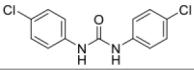	1,3-bis(4-chlorophenyl)urea	281,14	96,90%	**^{1}H NMR** (200 MHz, DMSO-*d6*): δ 8.86 (s, 2H, N-H), 7.56 – 7.42 (m, 4H, H_{Ar}), 7.39 – 7.26 (m, 4H, H_{Ar}); **^{13}C NMR** (50 MHz, DMSO-*d6*): δ 152.34, 138.55 (2C), 128.63 (4C), 125.49 (2C), 119.82 (4C).	$[M+H]^+$ calc. for $C_{13}H_{11}Cl_2N_2S^+$, 281.02, found **281.11**
11	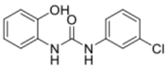	1-(3-chlorophenyl)-3-(2-hydroxyphenyl)urea	262,69		**^{1}H NMR** (400 MHz, DMSO-*d6*): δ 9.97 (s, 1H), 9.50 (s, 1H), 8.20 (s, 1H), 8.03 (d, *J* = 7.7 Hz, 1H, H_{Ar}), 7.73 (s, 1H), 7.29 (t, *J* = 8.0 Hz, 1H, H_{Ar}), 7.21 (d, *J* = 8.5 Hz, 1H, H_{Ar}), 7.00 (d, *J* = 7.8 Hz, 1H, H_{Ar}), 6.88 – 6.71 (m, 3H, H_{Ar}); **^{13}C NMR** (101 MHz, DMSO-*d6*): δ 152.35, 145.72, 141.54, 133.31, 130.42, 127.55, 122.04, 121.25, 119.19, 118.70, 117.22, 116.27, 114.45.	$[M+H]^+$ calc. for $C_{13}H_{11}Cl_2N_2S^+$, 281.02, found **281.11**
12	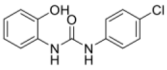	1-(4-chlorophenyl)-3-(2-hydroxyphenyl)urea	262,69	98,00%	**^{1}H NMR** (200 MHz, DMSO-*d6*): δ 9.98 (s, 1H), 9.45 (s, 1H), 8.19 (s, 1H), 8.09 – 7.96 (m, 1H, H_{Ar}), 7.48 (d, *J* = 8.8 Hz, 2H, H_{Ar}), 7.31 (d, *J* = 8.8 Hz, 2H, H_{Ar}), 6.91 – 6.67 (m, 3H, H_{Ar}); **^{13}C NMR** (50 MHz, DMSO-*d6*): δ 152.52, 145.77, 139.07, 128.73 (2C), 127.77, 125.24, 121.99, 119.43 (2C), 119.26, 118.76, 114.53.	$[M+H]^+$ calc. for $C_{13}H_{12}ClN_2O_2^+$, 263.05818; Found: **263.05823**
13	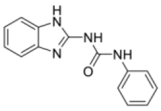	1-(1H-benzo[d]imidazol-2-yl)-3-phenylurea	252,28	96,40%	**^{1}H NMR** (200 MHz, DMSO-*d6*): δ 11.16 (br. s, 2H, 2N-H), 9.57 (s, 1H, N-H), 7.57 (d, *J* = 7.1 Hz, 2H, H_{Ar}), 7.48 – 7.21 (m, 4H, H_{Ar}), 7.17 – 6.90 (m, 3H, H_{Ar}); **^{13}C NMR** (50 MHz, DMSO-*d6*): δ 154.04, 148.95, 139.36, 135.03 (2C), 128.83 (2C), 122.32, 120.95 (2C), 118.55 (2C), 112.88 (2C).	$[M+H]^+$ calc. for $C_{14}H_{13}N_4O^+$, 253.11, found 253.13
14	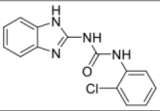	1-(1H-benzo[d]imidazol-2-yl)-3-(2-chlorophenyl)urea	286,72	98,20%	**^{1}H NMR** (200 MHz, DMSO-*d6*): δ (ppm) : 7.03-7.15 (m, 3H, H_{Ar}), 7.29-7.43 (m, 3H, H_{Ar}), 7.51 (dd, *J* = 8 Hz, *J* = 2 Hz, 1H, H_{Ar}), 8.27 (d, *J* = 8 Hz, 1H, H_{Ar}), 10.21 (s, 1H, N-H), 11.28 (s, 2H, N-H).	$[M+H]^+$, calc. for $C_{14}H_{12}ClN_4O^+$ 287.07, found 287.06
15	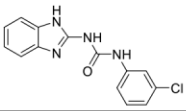	1-(1H-benzo[d]imidazol-2-yl)-3-(3-chlorophenyl)urea	286,72	98,00%	**^{1}H NMR** (200 MHz, DMSO-*d6*): δ 11.44 (br. s, 2H, 2N-H), 9.61 (s, 1H, N-H), 7.90 (t, *J* = 1.9 Hz, 1H, H_{Ar}), 7.47 – 7.24 (m, 4H, H_{Ar}), 7.14 – 6.95 (m, 3H, H_{Ar}); **^{13}C NMR** (50 MHz, DMSO-*d6*): δ 112.2 (2C), 116.8 (2C), 117.7 (2C), 121.3, 121.5 130.3 (2C), 133.1 (2C), 141.3 150.1.	ESI (m/z): $[M+H]^+$ for $C_{14}H_{12}ClN_4O^+$ 287.07, found 287.13
16	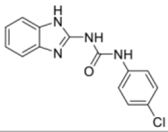	1-(1H-benzo[d]imidazol-2-yl)-3-(4-chlorophenyl)urea	286,72	96,00%	**^{1}H NMR** (200 MHz, DMSO-*d6*): δ 11.33 (s, 2H, 2N-H), 9.61 (s, 1H, N-H), 7.63 (d, *J* = 8.6 Hz, 2H, H_{Ar}), 7.34 (d, *J* = 6.1 Hz, 4H, H_{Ar}), 7.07 (dd, *J* = 4.6, 3.4 Hz, 2H, H_{Ar}); **^{13}C NMR** (50 MHz, DMSO-*d6*): δ 149.68, 138.79, 138.62, 133.86, 128.58 (2C), 125.51, 121.12 (2C), 119.95 (2C), 119.75, 112.45 (2C).	ESI (m/z): $[M+H]^+$ calc. for $C_{14}H_{12}ClN_4O^+$, 287.07, found 287.13
17	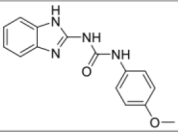	1-(1H-benzo[d]imidazol-2-yl)-3-(4-methoxyphenyl)urea	282,3	97,90%	**^{1}H NMR** (200 MHz, DMSO-*d6*): δ 10.99 (br. s, 2H, 2N-H), 9.46 (s, 1H, N-H), 7.47 (d, *J* = 8.7 Hz, 2H, H_{Ar}), 7.37 (dd, *J* = 5.5, 2.9 Hz, 2H, H_{Ar}), 7.05 (dd, *J* = 5.3, 2.9 Hz, 2H, H_{Ar}), 6.90 (d, *J* = 8.7 Hz, 2H, H_{Ar}), 3.73 (s, 3H, OCH3). **^{13}C NMR** (50 MHz, DMSO-*d6*): δ 154.97, 153.62, 148.86, 135.58 (2C), 132.24, 120.95 (2C), 120.52 (2C), 114.10 (2C), 113.12 (2C), 55.21.	$[M+H]^+$ calc. for $C_{15}H_{15}N_4O_2^+$, 283.11895; Found: **283.11902**
18	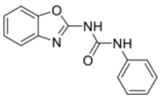	1-(benzo[d]oxazol-2-yl)-3-phenylurea	253,26	98,30%	**^{1}H NMR** (200 MHz, DMSO-*d6*): δ (ppm): 6.96 (t, *J* = 7 Hz, 1H, H_{Ar}), 7.27 (t, *J* = 7 Hz, 2H, H_{Ar}), 7.35-7.55 (m, 6H, H_{Ar}), 8.73 (s, 1H, N-H); **^{13}C NMR** (50 MHz, DMSO-*d6*): δ (ppm): 118.5 (2C), 122.0, 129.0 (2C), 129.1, 129.1 (2C), 129.2 (2C), 135.0, 140.0, 149.2, 152.9.	$[M+H-NHCO]^+$ calc. for $C_{13}H_{11}N_2O^+$, 211.09, found 211.27

19	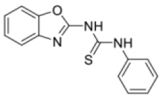	1-(benzo[d]oxazol-2-yl)-3-phenylthiourea	269,32	98,40%	**^{1}H NMR** (200 MHz, DMSO-*d6*): δ (ppm) : 7.03 (t, *J* = 8 Hz, 1H, H$_{Ar}$), 7.16 (td, *J* = 8 Hz, *J* = 2 Hz, 1H, H$_{Ar}$), 7.22 (td, *J* = 8 Hz, *J* = 2 Hz, 1H, H$_{Ar}$), 7.37 (t, *J* = 8 Hz, 2H, H$_{Ar}$), 7.47 (t, *J* = 8 Hz, 2H, H$_{Ar}$), 7.76 (d, *J* = 8 Hz, 2H, H$_{Ar}$), 10.62 (s, 1H, N-H). **^{13}C NMR** (50 MHz, DMSO-*d6*): δ 109.0, 116.6, 117.5 (2C), 121.7, 122.1, 124.0, 129.0 (2C), 133.1, 138.7, 142.4, 147.0, 158.0.	[M+H-NHCS]$^+$ calc. for $C_{13}H_{11}N_2O^+$, 211.09, found 211.27
20	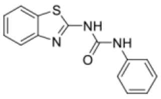	1-(benzo[d]thiazol-2-yl)-3-phenylurea	269,32	95,40%	**^{1}H NMR** (200 MHz, DMSO-*d6*): δ 10.88 (br. s, 1H, N-H), 9.19 (s, 1H, N-H), 7.91 (d, *J* = 7.1 Hz, 1H, H$_{Ar}$), 7.65 (d, *J* = 7.8 Hz, 1H, H$_{Ar}$), 7.55 (d, *J* = 1.2 Hz, 2H, H$_{Ar}$), 7.45 – 7.29 (m, 3H, H$_{Ar}$), 7.29 – 7.20 (m, 1H, H$_{Ar}$), 7.06 (t, *J* = 7.3 Hz, 1H, H$_{Ar}$); **^{13}C NMR** (50 MHz, DMSO-*d6*): δ 159.81, 152.29, 147.93, 138.56, 131.17, 128.97 (2C), 125.99, 122.99, 122.94, 121.54, 119.30, 118.89 (2C).	[M+H]$^+$ calc. for $C_{14}H_{12}N_3OS^+$, 270.06956; Found: **270.06961**
21	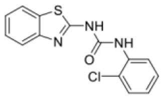	1-(benzo[d]thiazol-2-yl)-3-(2-chlorophenyl)urea	303,76	95,20%	**^{1}H NMR** (200 MHz, DMSO-*d6*): δ 11.46 (br. s, 1H, N-H), 9.14 (br. s, 1H, N-H), 8.18 (d, *J* = 8.1 Hz, 1H, H$_{Ar}$), 7.94 (d, *J* = 7.2 Hz, 1H, H$_{Ar}$), 7.69 (d, *J* = 8.1 Hz, 1H, H$_{Ar}$), 7.52 (d, *J* = 7.4 Hz, 1H, H$_{Ar}$), 7.46 – 7.21 (m, 3H, H$_{Ar}$), 7.12 (t, *J* = 7.2 Hz, 1H, H$_{Ar}$); **^{13}C NMR** (50 MHz, DMSO-*d6*): δ 159.31, 151.50, 148.93, 134.93, 131.33, 129.39, 127.76, 126.03, 124.48, 123.10, 122.74, 121.71, 121.55, 119.95.	[M+H]$^+$ calc. for $C_{14}H_{11}ClN_3OS^+$, 304.03059; Found: **304.03064**
22	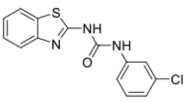	1-(benzo[d]thiazol-2-yl)-3-(3-chlorophenyl)urea	303,76	99,40%	**^{1}H NMR** (200 MHz, DMSO-*d6*): δ 11.17 (br. s, 1H, N-H), 9.41 (s, 1H, N-H), 7.90 (d, *J* = 7.3 Hz, 1H, H$_{Ar}$), 7.76 (d, *J* = 1.9 Hz, 1H, H$_{Ar}$), 7.63 (d, *J* = 7.8 Hz, 1H, H$_{Ar}$), 7.46 – 7.30 (m, 3H, H$_{Ar}$), 7.25 (td, *J* = 7.7, 1.2 Hz, 1H, H$_{Ar}$), 7.10 (dt, *J* = 7.1, 1.9 Hz, 1H, H$_{Ar}$); **^{13}C NMR** (50 MHz, DMSO-*d6*): δ 160.32, 153.03, 146.80, 140.27, 133.36, 130.70, 130.44, 126.05, 122.95, 122.50, 121.62, 118.71, 118.25, 117.28.	[M+H]$^+$ calc. for $C_{14}H_{11}ClN_3OS^+$, 304.03059; Found: **304.0306**
23	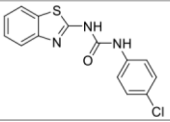	1-(benzo[d]thiazol-2-yl)-3-(4-chlorophenyl)urea	303,76	100,00%	**^{1}H NMR** (400 MHz, DMSO-*d6*): δ 10.97 (br. s, 1H, N-H), 9.33 (s, 1H, N-H), 7.90 (d, *J* = 7.8 Hz, 1H, H$_{Ar}$), 7.64 (d, *J* = 7.7 Hz, 1H, H$_{Ar}$), 7.57 (d, *J* = 8.7 Hz, 2H, H$_{Ar}$), 7.43 – 7.34 (m, 3H, H$_{Ar}$), 7.28 – 7.21 (m, 1H, H$_{Ar}$); **^{13}C NMR** (50 MHz, DMSO-*d6*): δ 160.11, 152.74, 147.16, 137.66, 130.83, 128.77, 126.54, 126.04, 122.95, 121.60, 120.39, 118.95.	[M+H]$^+$ calc. for $C_{14}H_{11}ClN_3OS^+$, 304.03059; Found: **304.03076**
24	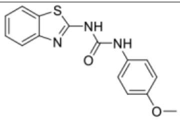	1-(benzo[d]thiazol-2-yl)-3-(4-methoxyphenyl)urea	299,35	97,30%	**^{1}H NMR** (200 MHz, DMSO-*d6*): δ 10.81 (s, 1H, N-H), 9.00 (s, 1H, N-H), 7.90 (d, *J* = 7.8 Hz, 1H, H$_{Ar}$), 7.65 (d, *J* = 7.9 Hz, 1H, H$_{Ar}$), 7.50 – 7.33 (m, 3H, H$_{Ar}$), 7.23 (t, *J* = 7.5 Hz, 1H, H$_{Ar}$), 6.92 (d, *J* = 8.9 Hz, 2H, H$_{Ar}$), 3.73 (s, 3H, OCH3); **^{13}C NMR** (50 MHz, DMSO-*d6*): δ 159.85, 155.32, 152.26, 148.23, 131.45, 131.28, 125.95, 122.83, 121.49, 120.83 (2C), 119.37, 114.13 (2C), 55.19.	[M+H]$^+$ calc. for $C_{15}H_{14}N_3O_2S^+$, 300.08012; Found: **300.08023**
25	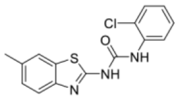	1-(2-chlorophenyl)-3-(6-methylbenzo[d]thiazol-2-yl)urea	317,79	99,20%	**^{1}H NMR** (200 MHz, DMSO-*d6*): δ 11.39 (s, 1H, N-H), 9.14 (s, 1H, N-H), 8.18 (dd, *J* = 8.3, 1.4 Hz, 1H, H$_{Ar}$), 7.73 (s, 1H, H$_{Ar}$), 7.63 – 7.45 (m, 2H, H$_{Ar}$), 7.42 – 7.30 (m, 1H, H$_{Ar}$), 7.22 (dd, *J* = 8.3, 1.2 Hz, 1H, H$_{Ar}$), 7.12 (td, *J* = 7.6, 1.5 Hz, 1H, H$_{Ar}$), 2.40 (s, 3H, CH_3); **^{13}C NMR** (50 MHz, DMSO-*d6*): δ 158.38, 151.37, 146.74, 134.91, 132.41, 131.37, 129.27, 127.64, 127.18, 124.24, 122.52, 121.48, 121.12, 119.52, 20.83.	[M+H]$^+$ calc. for $C_{15}H_{13}ClN_3OS^+$, 318.04624; Found: **318.04630**
26	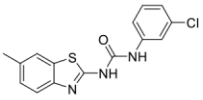	1-(3-chlorophenyl)-3-(6-methylbenzo[d]thiazol-2-yl)urea	317,79	95,50%	**^{1}H NMR** (400 MHz, DMSO-*d6*): δ 11.06 (br. s, 1H, N-H), 9.37 (s, 1H, N-H), 7.76 (s, 1H, H$_{Ar}$), 7.68 (s, 1H, H$_{Ar}$), 7.51 (d, *J* = 7.4 Hz, 1H, H$_{Ar}$), 7.42 – 7.30 (m, 2H, H$_{Ar}$), 7.20 (d, *J* = 7.7 Hz, 1H, H$_{Ar}$), 7.09 (d, *J* = 6.9 Hz, 1H, H$_{Ar}$), 2.38 (s, 3H, CH_3); **^{13}C NMR** (50 MHz, DMSO-*d6*): δ 159.52, 152.91, 144.86, 140.33, 133.42, 132.36, 130.89, 130.39, 127.25, 122.44, 121.29, 118.41, 118.23, 117.23, 20.90.	[M+H]$^+$ calc. for $C_{15}H_{13}ClN_3OS^+$, 318.04624; Found: **318.04626**
27	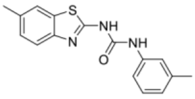	1-(6-methylbenzo[d]thiazol-2-yl)-3-(m-tolyl)urea	297,38	99,60%	**^{1}H NMR** (200 MHz, DMSO-*d6*): δ 10.75 (br. s, 1H, N-H), 9.08 (s, 1H, N-H), 7.70 (s, 1H, H$_{Ar}$), 7.53 (d, *J* = 8.1 Hz, 1H, H$_{Ar}$), 7.35 (s, 1H, H$_{Ar}$), 7.33 – 7.15 (m, 3H, H$_{Ar}$), 6.87 (d, *J* = 7.2 Hz, 1H, H$_{Ar}$), 2.39 (s, 3H, CH_3), 2.30 (s, 3H, CH_3); **^{13}C NMR** (50 MHz, DMSO-*d6*): δ 158.91, 152.09, 146.09, 138.51, 138.24, 132.26, 131.34, 128.77, 127.17, 123.68, 121.19, 119.30, 119.04, 115.97, 21.18, 20.89.	[M+H]$^+$ calc. for $C_{16}H_{16}N_3OS^+$, 298.10086; Found: **298.10092**
28	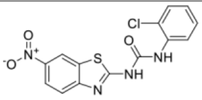	1-(2-chlorophenyl)-3-(6-nitrobenzo[d]thiazol-2-yl)urea	348,76	97,70%	**^{1}H NMR** (400 MHz, DMSO-*d6*): δ 11.79 (s, 1H, N-H), 8.93 (s, 1H, N-H), 8.90 (d, *J* = 2.4 Hz, 1H, H$_{Ar}$), 8.17 (dd, *J* = 8.9, 2.4 Hz, 1H, H$_{Ar}$), 8.12 (dd, *J* = 8.3, 1.2 Hz, 1H, H$_{Ar}$), 7.74 (d, *J* = 8.9 Hz, 1H, H$_{Ar}$), 7.47 (dd, *J* = 8.0, 1.3 Hz, 1H, H$_{Ar}$), 7.36 – 7.30 (m, 1H, H$_{Ar}$), 7.10 (td, *J* = 7.9, 1.4 Hz, 1H, H$_{Ar}$); **^{13}C NMR** (101 MHz, DMSO-*d6*): δ 164.50, 153.86, 151.28, 142.48, 134.53, 132.19, 129.37, 127.74, 124.73, 122.84, 121.66, 121.63, 119.80, 118.58.	[M+H]$^+$ calc. for $C_{14}H_{10}ClN_4O_3S^+$, 349.01567; Found: **349.01569**
29	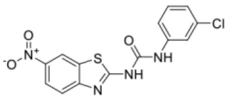	1-(3-chlorophenyl)-3-(6-nitrobenzo[d]thiazol-2-yl)urea	348,76	100,00%	**^{1}H NMR** (400 MHz, DMSO-*d6*): δ 11.44 (s, 1H, N-H), 9.41 (s, 1H, N-H), 8.95 (d, *J* = 1.9 Hz, 1H, H$_{Ar}$), 8.23 (dd, *J* = 8.9, 2.4 Hz, 1H, H$_{Ar}$), 7.76 (d, *J* = 8.8 Hz, 1H, H$_{Ar}$), 7.72 (s, 1H, H$_{Ar}$), 7.41 – 7.32 (m, 2H, H$_{Ar}$), 7.12 (dd, *J* = 8.9, 1.7 Hz, 1H, H$_{Ar}$); **^{13}C NMR** (101 MHz, DMSO-*d6*): δ 164.86, 153.40, 152.37, 142.54, 139.76, 133.29, 131.87, 130.57, 122.93, 121.85, 119.20, 118.73, 118.42, 117.53.	[M+H]$^+$ calc. for $C_{14}H_{10}ClN_4O_3S^+$, 349.01567; Found: **349.01569**
30	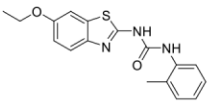	1-(6-ethoxybenzo[d]thiazol-2-yl)-3-(o-tolyl)urea	327,40	97,00%	**^{1}H NMR** (200 MHz, DMSO-*d6*): δ 10.97 (br. s, 1H, N-H), 8.64 (s, 1H, N-H), 7.86 (d, *J* = 7.7 Hz, 1H, H$_{Ar}$), 7.63 – 7.42 (m, 2H, H$_{Ar}$), 7.20 (t, *J* = 8.5 Hz, 2H, H$_{Ar}$), 7.09 – 6.89 (m, 2H, H$_{Ar}$), 4.05 (dd, *J* = 13.7, 6.6 Hz, 2H, CH_2), 2.28 (s, 3H, CH_3), 1.34 (t, *J* = 6.8 Hz, 3H, CH_3); **^{13}C NMR** (50 MHz, DMSO-*d6*): δ 157.52, 154.98, 151.65, 143.04, 136.36, 132.58, 130.37, 127.97, 126.36, 123.64, 121.13, 120.41, 114.76, 105.54, 63.58, 17.81, 14.74.	[M+H]$^+$ calc. for $C_{17}H_{18}N_3O_2S^+$, 328.11142; Found: **328.11154**
31	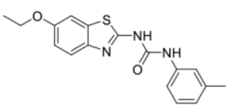	1-(6-ethoxybenzo[d]thiazol-2-yl)-3-(m-tolyl)urea	327,40	97,50%	**^{1}H NMR** (200 MHz, DMSO-*d6*): δ 10.65 (br. s, 1H, N-H), 9.05 (s, 1H, N-H), 7.54 (d, *J* = 8.8 Hz, 1H, H$_{Ar}$), 7.50 (d, *J* = 2.5 Hz, 1H, H$_{Ar}$), 7.38 – 7.15 (m, 3H, H$_{Ar}$), 6.96 (dd, *J* = 8.8, 2.6 Hz, 1H, H$_{Ar}$), 6.87 (d, *J* = 6.9 Hz, 1H, H$_{Ar}$), 4.05 (q, *J* = 6.9 Hz, 2H, CH_2), 2.30 (s, 3H, CH_3), 1.34 (t, *J* = 7.0 Hz, 3H, CH_3); **^{13}C NMR** (50 MHz, DMSO-*d6*): δ 157.57, 154.95, 151.86, 142.31, 138.46, 138.24, 132.49, 128.78, 123.65, 120.04, 119.25, 115.91, 114.79, 105.52, 63.59, 21.17, 14.73.	[M+H]$^+$ calc. for $C_{17}H_{18}N_3O_2S^+$, 328.11142; Found: **328.11151**
32	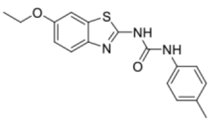	1-(6-ethoxybenzo[d]thiazol-2-yl)-3-(p-tolyl)urea	327,40	100,00%	**^{1}H NMR** (200 MHz, DMSO-*d6*): δ 10.63 (s, 1H, N-H), 9.03 (s, 1H, N-H), 7.54 (d, *J* = 8.8 Hz, 1H, H$_{Ar}$), 7.49 (d, *J* = 2.5 Hz, 1H, H$_{Ar}$), 7.39 (d, *J* = 8.4 Hz, 2H, H$_{Ar}$), 7.13 (d, *J* = 8.3 Hz, 2H, H$_{Ar}$), 6.96 (dd, *J* = 8.8, 2.6 Hz, 1H, H$_{Ar}$), 4.05 (q, *J* = 6.9 Hz, 2H, CH_2), 2.26 (s, 3H, CH_3), 1.34 (t, *J* = 7.0 Hz, 3H, CH_3); **^{13}C NMR** (50 MHz, DMSO-*d6*): δ 157.56, 154.94, 151.90, 142.41, 135.98, 132.52, 131.86, 129.34 (2C), 120.07, 118.84 (2C), 114.73, 105.53, 63.57, 20.39, 14.73.	[M+H]$^+$ calc. for $C_{17}H_{18}N_3O_2S^+$, 328.11142; Found: **328.11157**
33	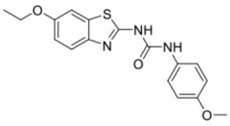	1-(6-ethoxybenzo[d]thiazol-2-yl)-3-(4-methoxyphenyl)urea	343,4	98,20%	**^{1}H NMR** (200 MHz, DMSO-*d6*): δ 10.61 (br. s, 1H, N-H), 8.95 (s, 1H, N-H), 7.53 (d, *J* = 8.8 Hz, 1H, H$_{Ar}$), 7.49 (d, *J* = 2.5 Hz, 1H, H$_{Ar}$), 7.46 – 7.33 (m, 2H, H$_{Ar}$), 7.01 – 6.85 (m, 3H, H$_{Ar}$), 4.04 (q, *J* = 6.9 Hz, 2H, CH_2), 3.73 (s, 3H, OCH_3), 1.34 (t, *J* = 6.9 Hz, 3H, CH_3); **^{13}C NMR** (50 MHz, DMSO-*d6*): δ 157.71, 155.25, 154.96, 152.03, 142.51, 132.58, 131.49, 120.72 (2C), 120.12, 114.67, 114.10 (2C), 105.53, 63.60, 55.17, 14.72.	[M+H]$^+$ calc. for $C_{17}H_{18}N_3O_3S^+$, 344.10634; Found: **344.10641**

Figure S1

A

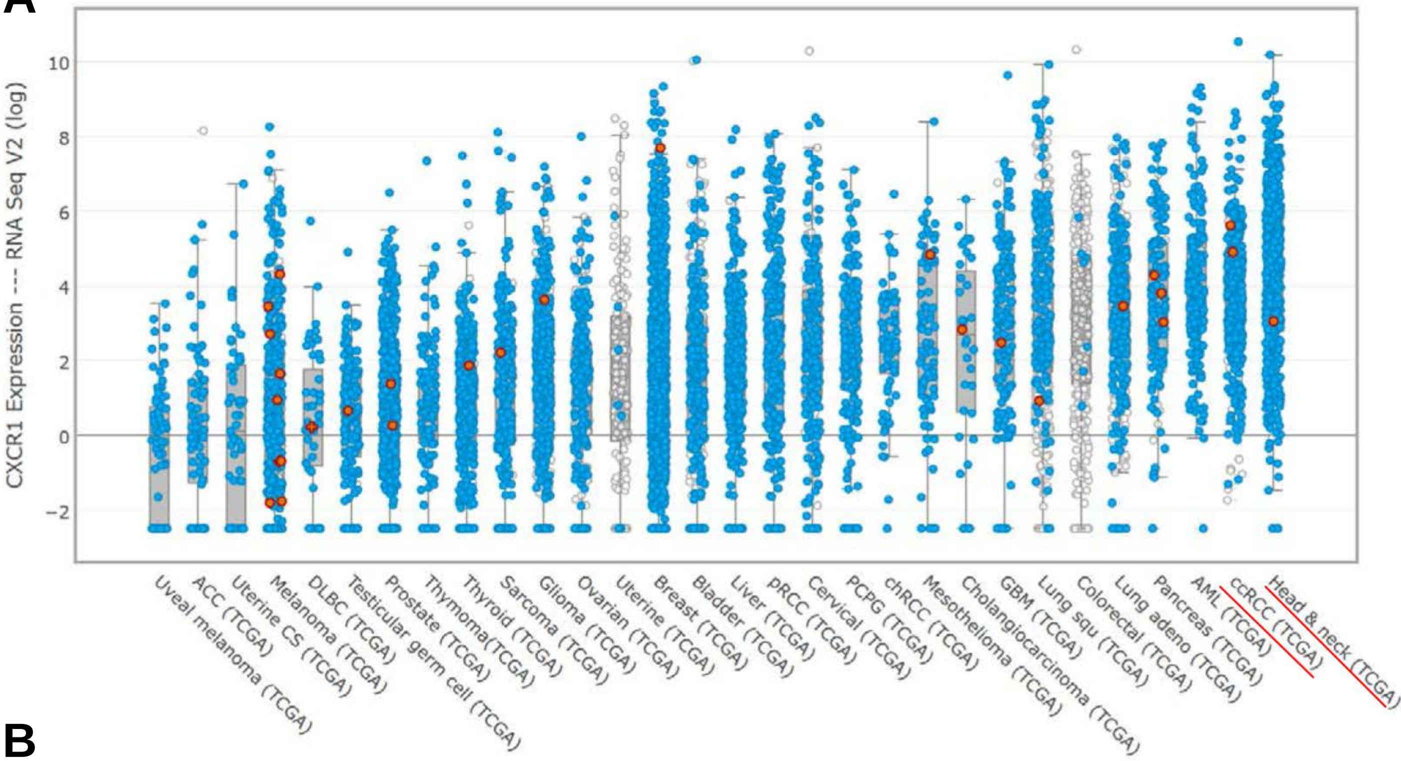

B

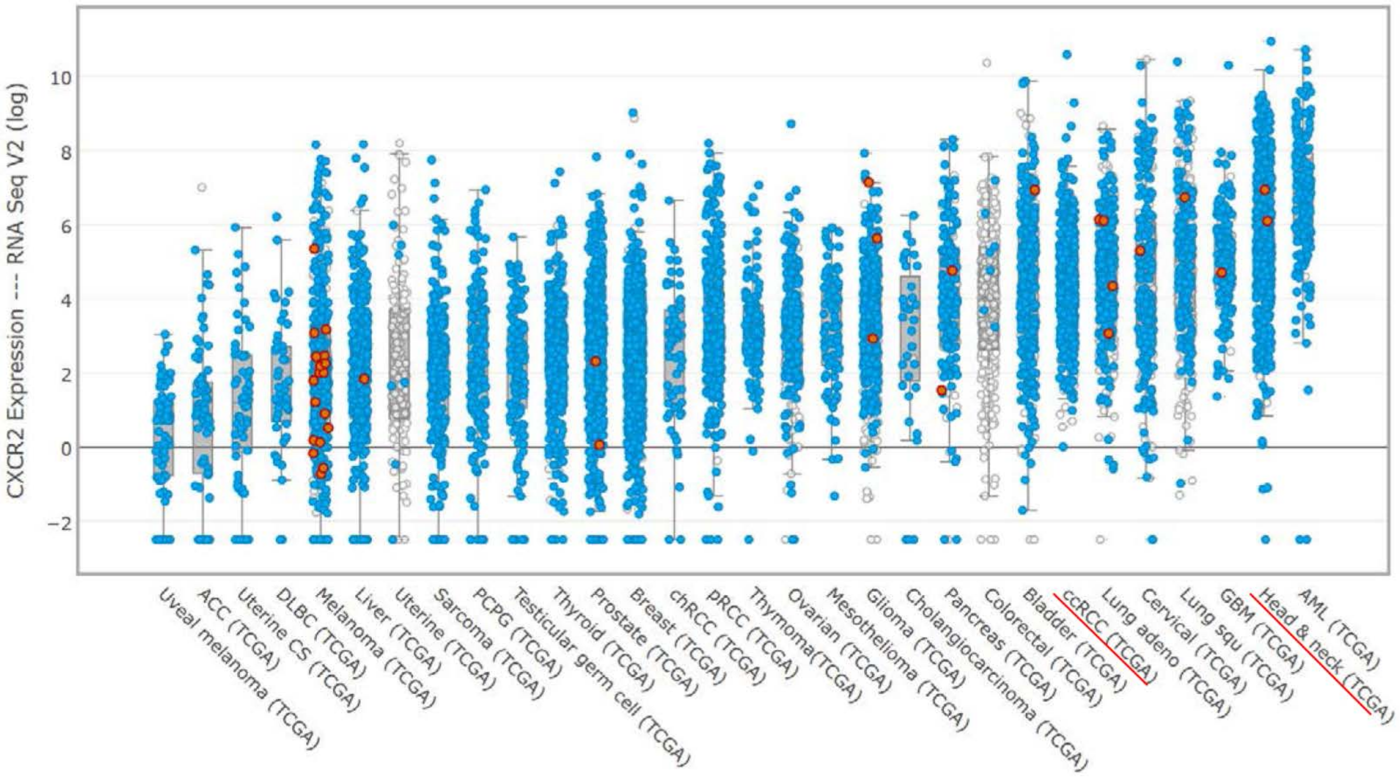

Figure S2: Dufies *et al*

RCC

HNSCC

A

p< 0.0001

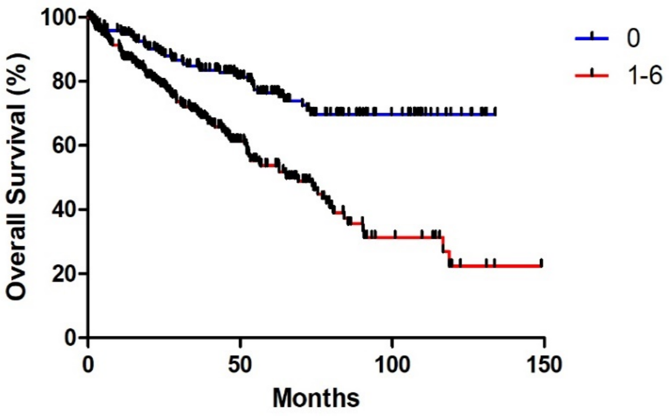

CXCL score	0	1-6
Patients nb	201	245
OS (Months)	NR	65

C

p=0.0043

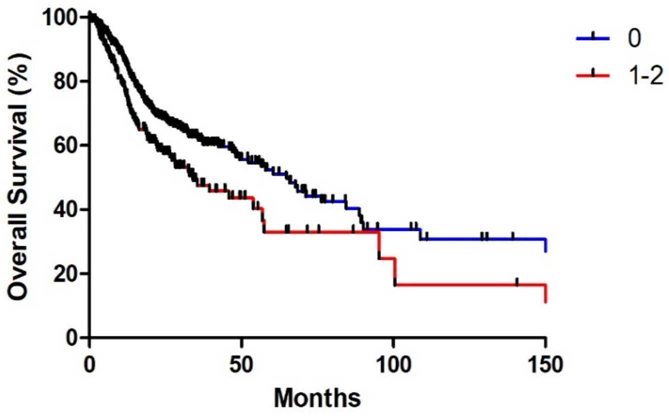

CXCL score	0	1-2
Patients nb	324	170
OS (Months)	65	34

B

p=0.0002

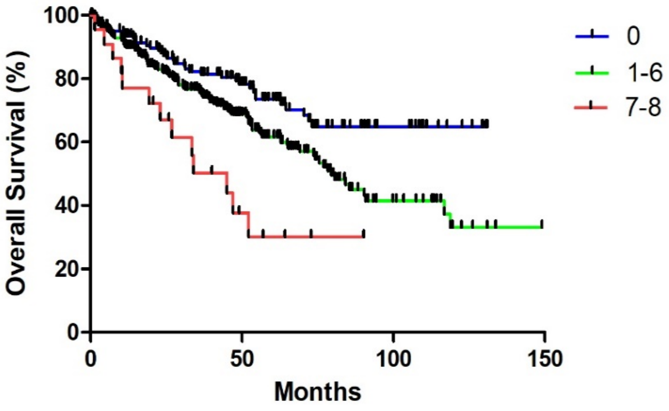

CXCL-CXCR score	0	1-6	7-8
Patients nb	150	274	22
OS (Months)	NR	80	45

D

p=0.0082

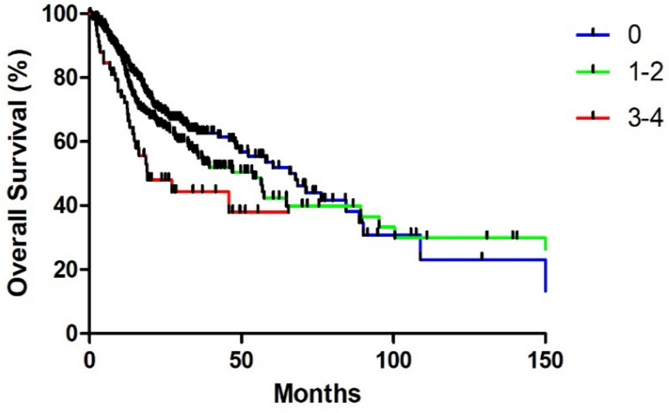

CXCL-CXCR score	0	1-2	3-4
Patients nb	221	214	59
OS (Months)	68	54	19

Figure S3, Dufies *et al*

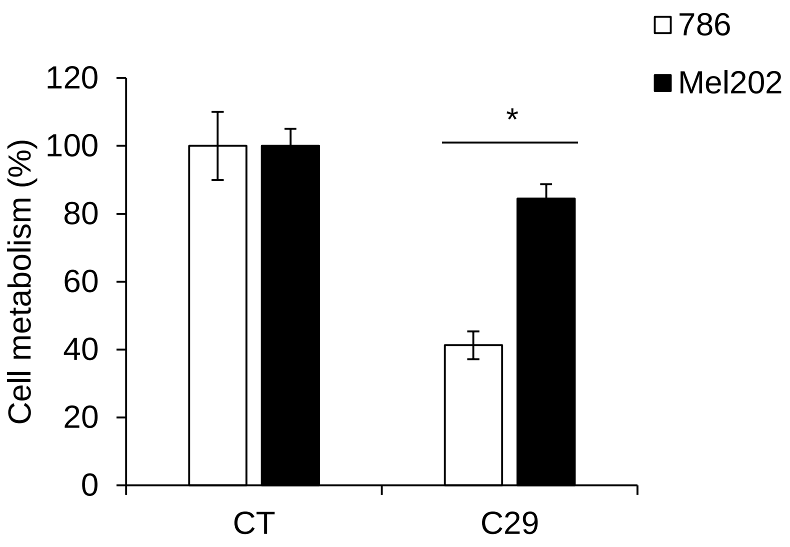

Figure S4: Dufies *et al*

A

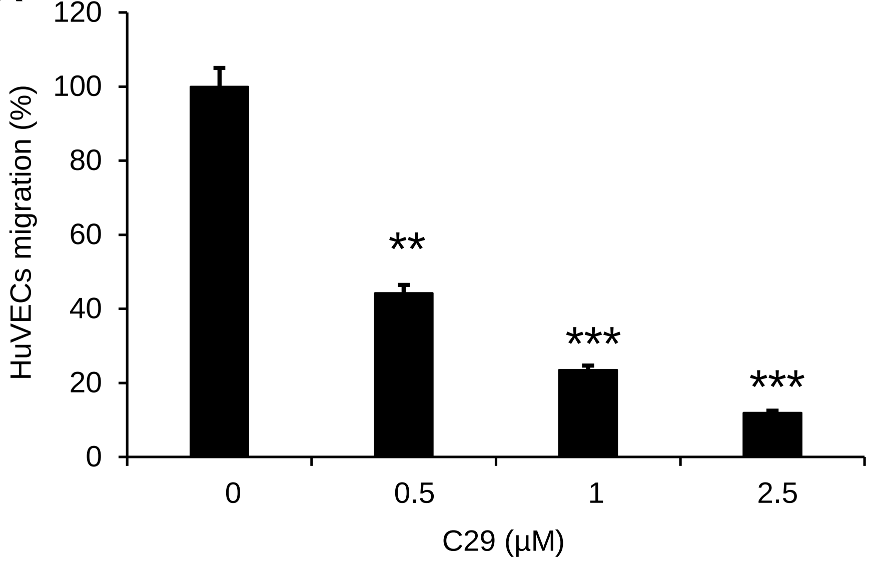

B

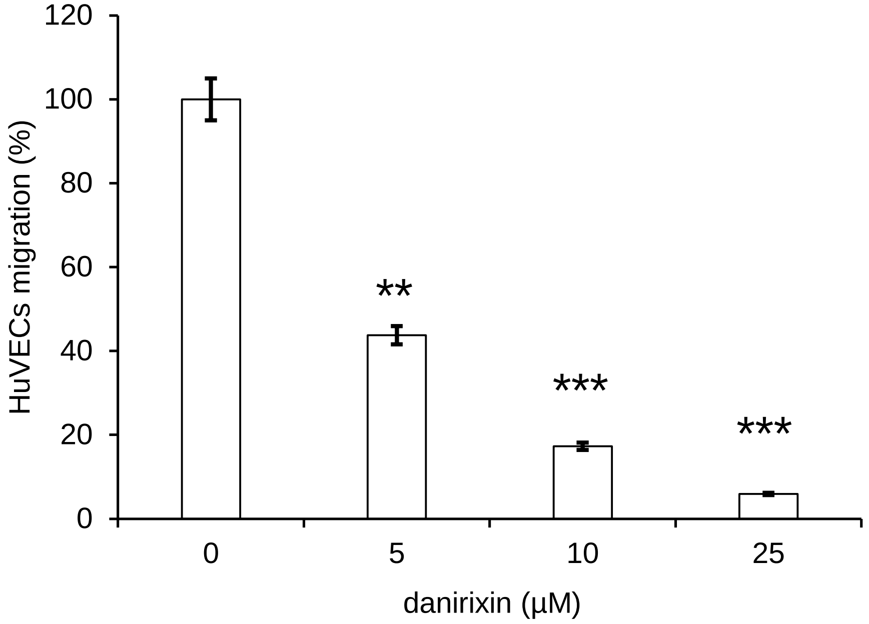

Figure S5: Dufies *et al*

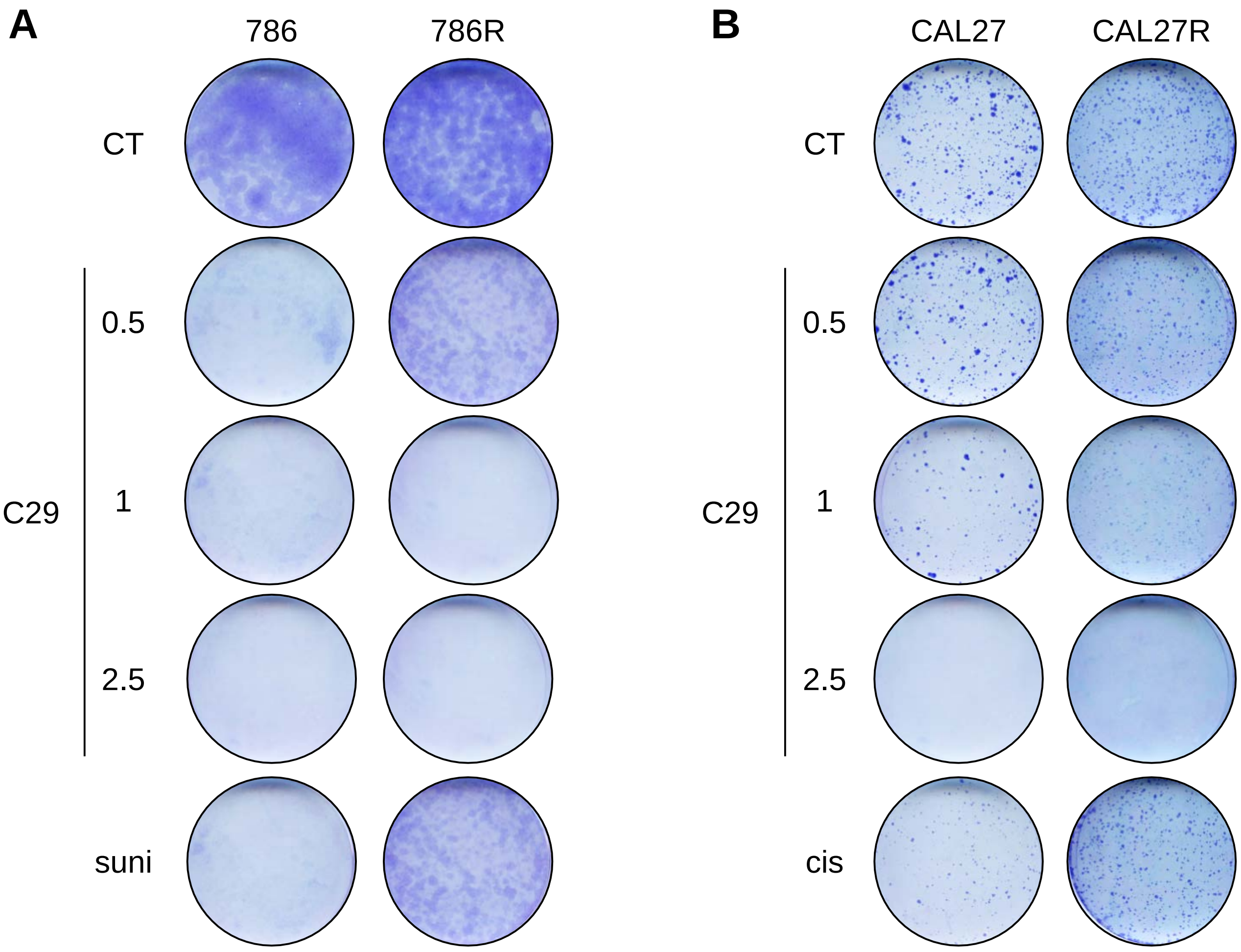

Figure S6: Dufies *et al*

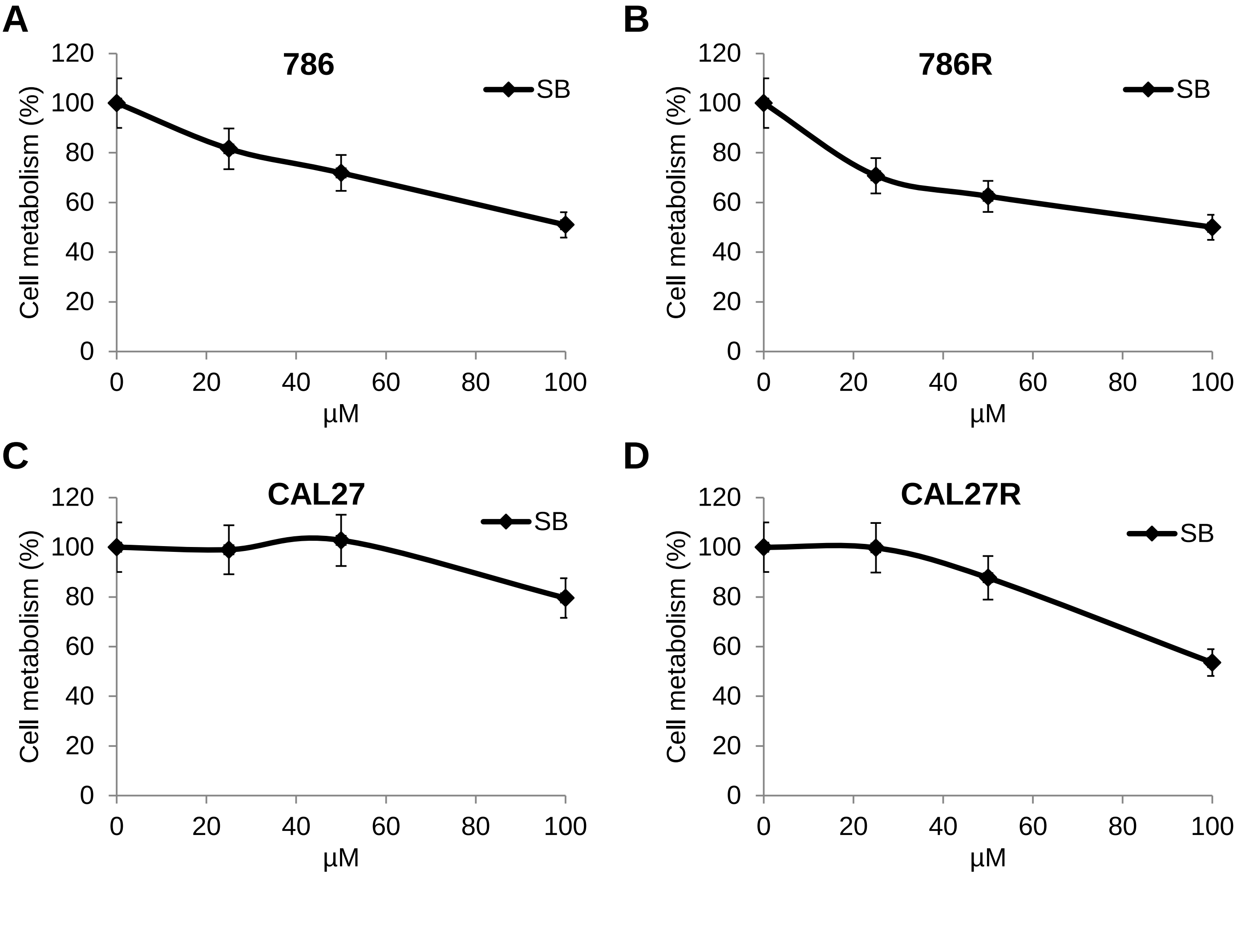

Figure S7: Dufies *et al*

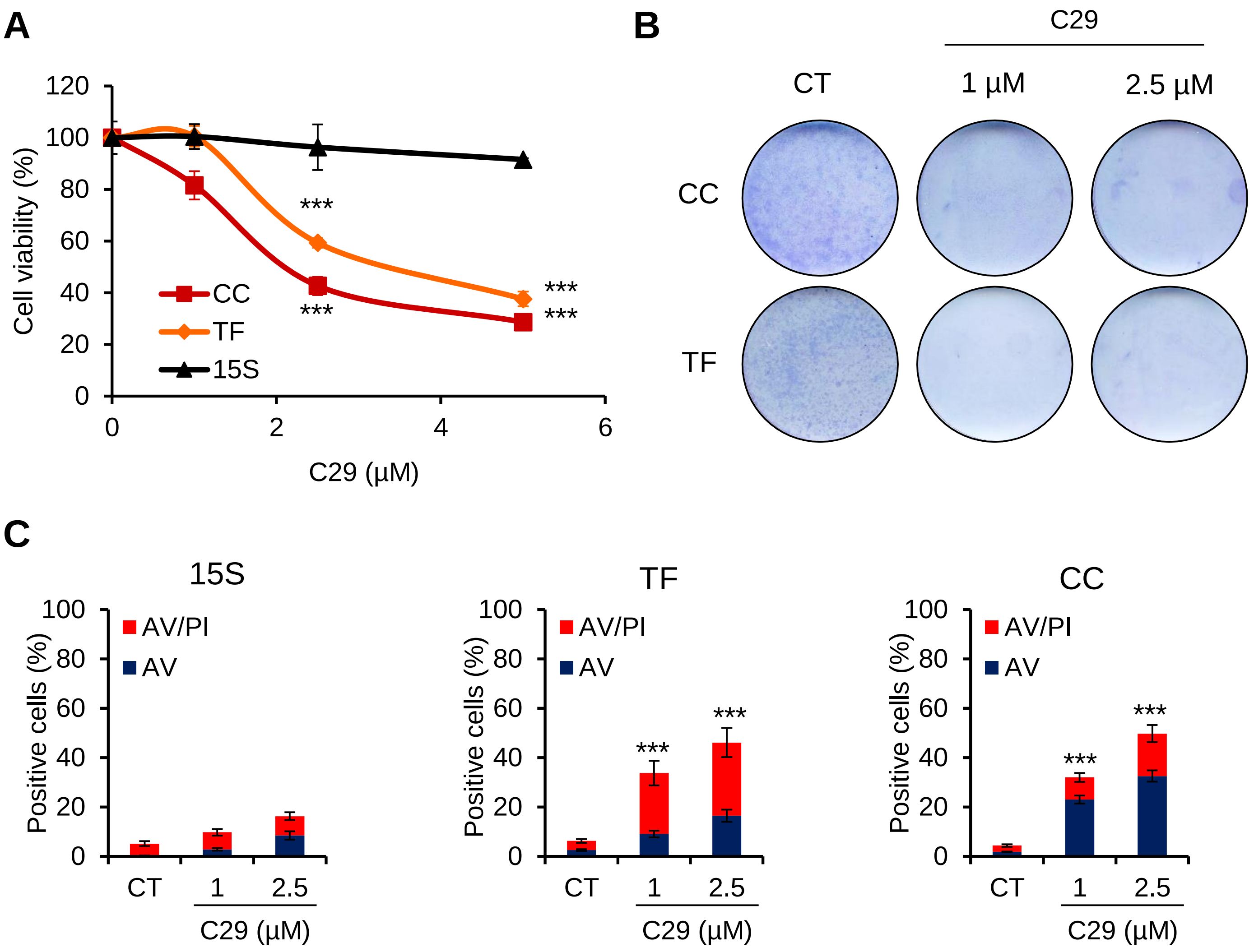

Figure S8: Dufies *et al*

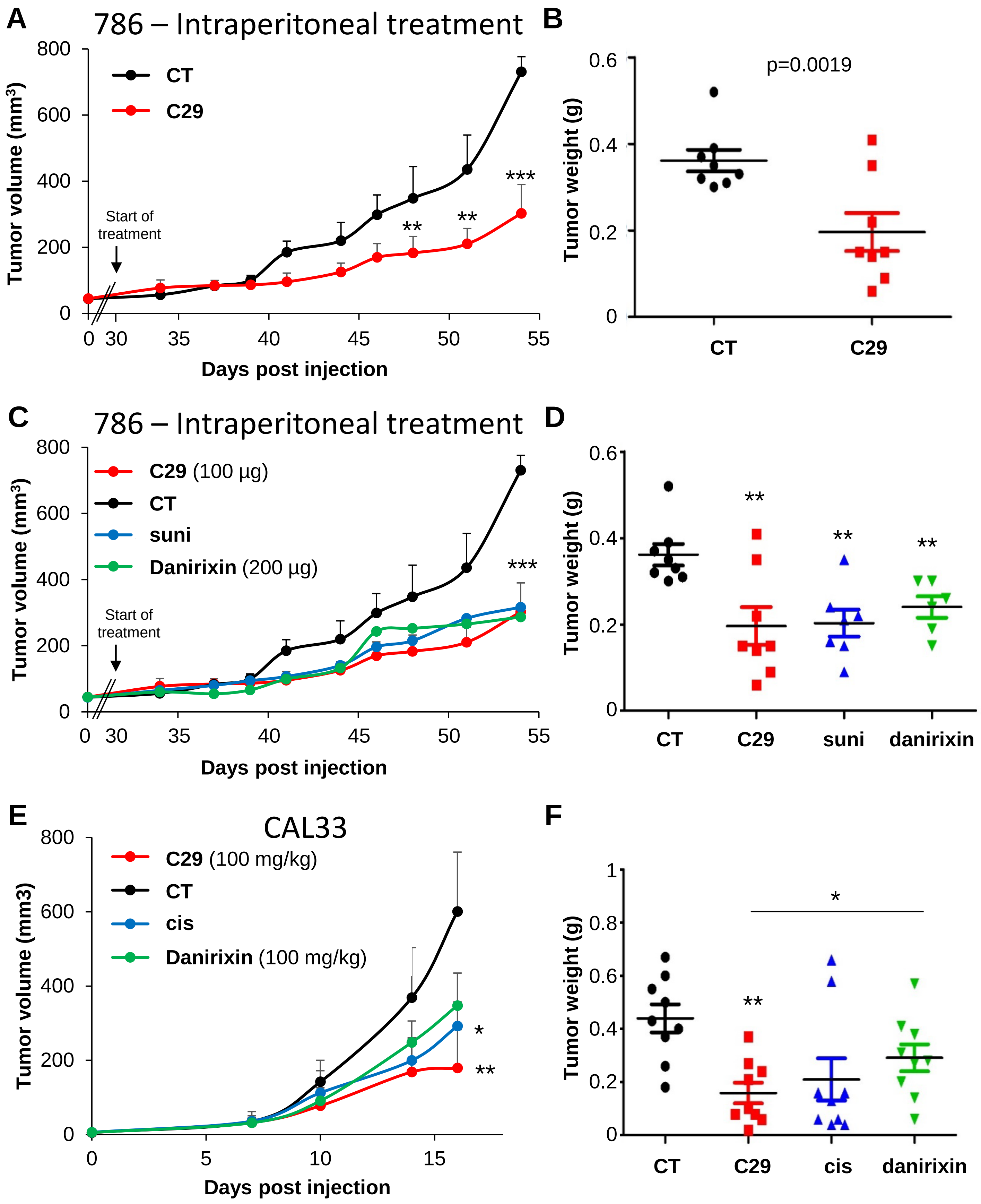

Figure S9: Dufies *et al*

A

	CT	C29	P Value
**Leucocytes (k/µl)**	3.774 ± 0.49	4.239 ± 0.60	ns
**Neutrophils (k/µl)**	0.7836 ± 0.12	0.6760 ± 0.11	ns
**Lymphocytes (k/µl)**	2.528 ± 0.29	2.943 ± 0.38	ns
**Monocytes (k/µl)**	0.2671 ± 0.04	0.2664 ± 0.04	ns
**Eosinophils (k/µl)**	0.1478 ± 0.03	0.1760 ± 0.04	ns
**Basophils (k/µl)**	0.05289 ± 0.01	0.07089 ± 0.02	ns
**Red blood cells (M/µl)**	8.514 ± 1.33	10.33 ± 0.98	ns
**Hemoglobin (g/dl)**	11.61 ± 1.04	14.67 ± 1.22	ns
**Hematocrit (%)**	21.56 ± 1.94	26.22 ± 2.45	ns
**Mean Corpuscular Volume (fl)**	111.4 ± 1.47	112.0 ± 1.054	ns
**Mean corpuscular hemoglobin (pg)**	31.42 ± 0.78	31.46 ± 0.79	ns
**Mean corpuscular hemoglobin concentration (g/dl)**	62.13 ± 1.98	61.87 ± 1.49	ns
**Platelets (k/µl)**	348.5 ± 83.56	375.7 ± 77.93	ns
**Mean platelet volume (fl)**	13.02 ± 0.34	12.81 ± 0.08	ns

B

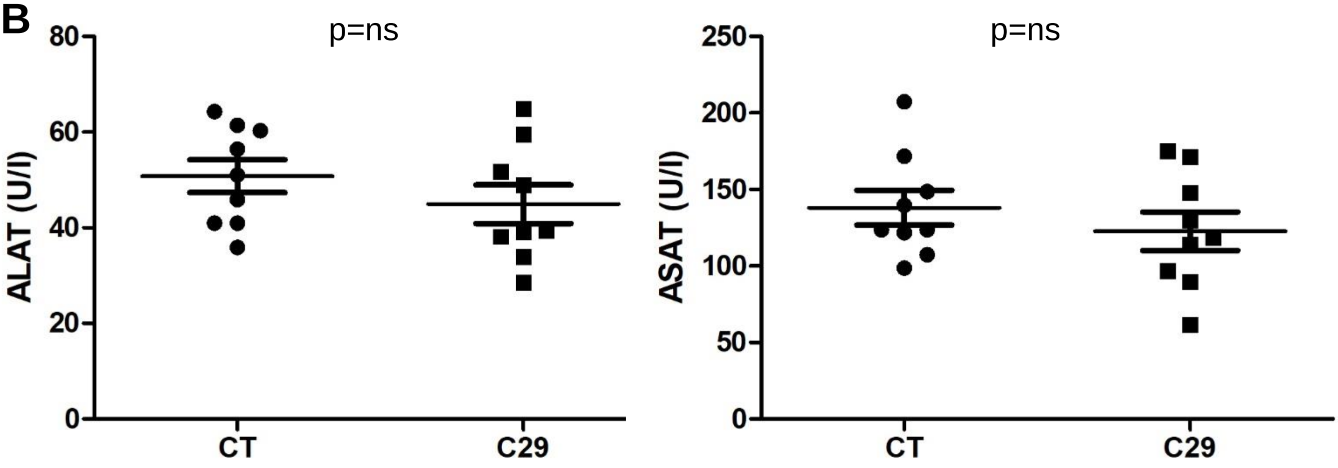

C

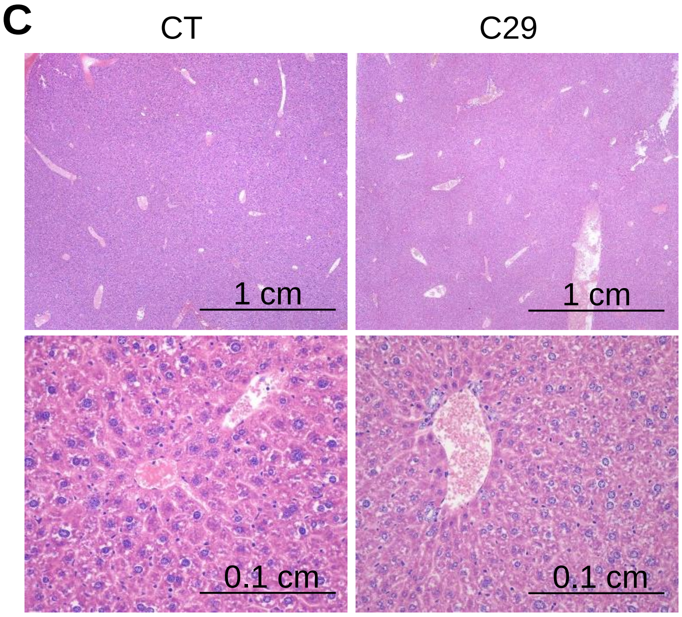

D

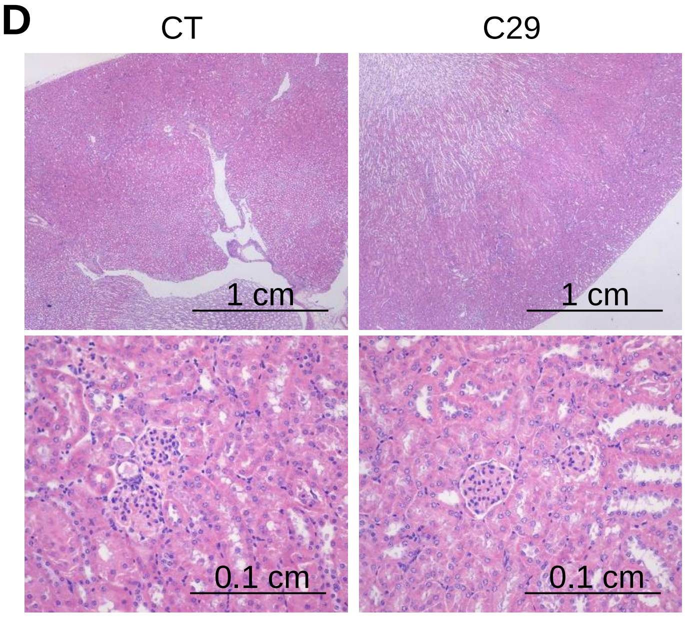

Figure S10: Dufies *et al*
